# Translational Barriers and AI‐Driven Challenges of Microfluidics‐Enabled Wearables and Implantable Systems in Personalized Medicine

**DOI:** 10.1002/advs.77296

**Published:** 2026-09-27

**Authors:** Ke Huang, Ching Yin Fong, Xin Huang, Bee Luan Khoo

**Affiliations:** ^1^ Department of Biomedical Engineering College of Biomedicine City University of Hong Kong Hong Kong China; ^2^ Hong Kong Center for Cerebro‐Cardiovascular Health Engineering (COCHE) Hong Kong China; ^3^ Institute of Digital Medicine City University of Hong Kong Hong Kong SAR China

**Keywords:** AI‐driven biomarker analytics, closed‐loop drug delivery, microfluidic, personalized medicine, wearable biosensors

## Abstract

Wearable and implantable microfluidic systems have progressed from laboratory prototypes toward translational clinical deployment, enabling continuous, minimally invasive sampling of dynamic biomarkers across sweat, saliva, tears, and interstitial fluid. However, existing reviews often address materials chemistry or device fabrication in isolation, obscuring the systemic path to clinical translation. This review establishes a cohesive, translation‐focused linear trajectory starting from foundational functional biomaterials and advanced fabrication techniques, transitioning into a performance benchmarking of diagnostics‐oriented systems and closed‐loop theranostic platforms. Through these vectors, we systematically evaluate how microfluidic transport, multiplexed molecular analytics, and autonomous therapeutic integration collectively drive the shift from passive tracking to adaptive intervention. Beyond physical hardware, we decode the integration of artificial intelligence (AI) across three precise pathways: sensor self‐calibration, multiplexed molecular decoding, and on‐device autonomous decision‐making. Finally, we critically examine the socio‐technical barriers to clinical translation, with a focus on how biofluid data heterogeneity and population baseline disparities propagate algorithmic bias. We propose that robust hardware interfaces, standardized validation benchmarks, and alignment with emerging regulatory frameworks are prerequisites for achieving equitable, responsible digital health protection.

## Graphical abstract

An integrative review of microfluidics‐enabled wearables and implantable systems reveals a single‐track translation pipeline, bridging functional biomaterials with clinical utility. Dynamic feedback loops driven by artificial intelligence advance diagnostics toward personalized closed‐loop theranostics. Critical evaluation of algorithmic bias and data heterogeneity establishes a responsible, system‐level framework to guide equitable deployment in digital health.

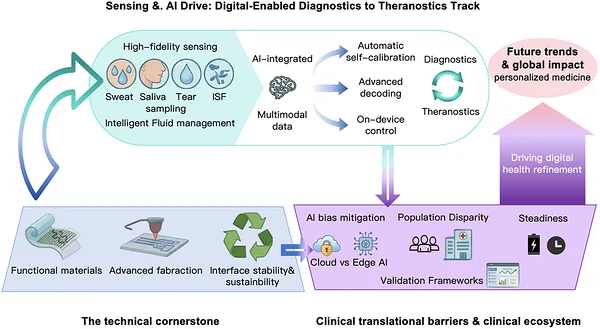

**Abbreviations**
µSHM: microfluidic mixer
3D: three‐dimensional
AI: Artificial Intelligence
ANNs: Artificial neural networks
AuNCs: gold nanocubes
CNN: Convolutional neural network
CV: cyclic voltammetry
DL: Deep learning
DMF: digital microfluidics
ECG: Electrocardiogram
EMA: European Medicines Agency
e‐skin: electronic skin
FDA: Food and Drug Administration FDA)
GPIO: general‐purpose input/output
GRU: Gated Recurrent Unit
H_2_O_2_
: hydrogen peroxide
HR: heart rate
IL‐6: interleukin‐6
ISF: interstitial fluid
LOC: lab‐on‐a‐chip
LSTM: Long Short Short‐Term Memory
MAC: multiply‐add
ML: Machine learning
NSCLC: non‐small cell lung cancer
OoC: organ‐on‐a‐chip
PCL: polycaprolactone
PDMS: polydimethylsiloxane
PLA: Polylactic acid
PMMA: Polymethyl methacrylate
RPA: recombinase polymerase amplification
SERS: surface‐enhanced Raman scattering
SM: separation membrane
STFT: short‐time Fourier Transform
TM: transport membrane
WP: wax plug

## 1 Introduction

The escalating global burden of chronic diseases, such as cardiovascular disorders, diabetes, and cancer, coupled with demographic aging, is exerting unsustainable pressure on traditional healthcare models [1]. These conditions, characterized by their progressive nature, demand a paradigm shift from episodic, reactive care to continuous, predictive, and personalized management [2]. While advances in medical care have extended life expectancy, they have also contributed to a growing elderly demographic that is disproportionately affected by chronic conditions, further exacerbating clinical and economic strains. The associated costs extend beyond direct medical expenditures, such as recurring clinical visits and long‐term pharmacotherapy, to encompass substantial indirect burdens, such as caregiver expenses and productivity loss. This economic reality underscores an urgent need for innovative, scalable, and efficient healthcare solutions.

In response to this pressing clinical demand, the past decade has witnessed the remarkable emergence and rapid maturation of wearable and implantable biomedical technologies, transitioning from passive personal gadgets into clinical‐grade physiological diagnostic networks [3]. The successful development of wearable and implantable technologies, which began with simple step counters and heart rate (HR) monitors for long‐term usage, has evolved into a sophisticated ecosystem of devices capable of capturing a rich array of physiological data [4, 5]. demonstrating that continuous physiological data can reliably inform diagnostic and therapeutic decisions. This shift empowers individuals and clinicians with unprecedented insights, moving diagnostics from the confines of the laboratory to the point of need [6].

Besides, the growing research in microelectronics and materials science is providing the essential building blocks for the emergence of wearable and implantable technologies. Microfluidics has emerged as the enabling technology for wearable and implantable biochemical sensing, operating at a scale commensurate with biological processes [7, 8]. The miniaturised sensors and energy‐dense batteries enabled the design of devices that were not only small and light enough for all‐day wear or long‐term implantation but also capable of performing complex computational tasks within the human body [9, 10]. The biomimetic flexibility is no longer just an elegant improvement; it has become a fundamental requirement for both user comfort and data accuracy. The body's immune system is exquisitely tuned to identify and attack foreign materials, which is a process known as the foreign body response [11]. By facilitating the integration of micropatterned fluidic pathways with flexible electronics, microfluidics supports the development of highly conformable and functional devices. Therefore, the advancement of implantable devices has been closely linked to innovation in biomaterials [12]. The emergence of novel polymers, bioinert ceramics, and sophisticated hydrogel coatings designed to mimic native tissue has been crucial in creating a stable, biologically neutral interface [13,14]. It ensures that the device can function not just as a passive observer but as a part of the physiological landscape for years on end [15].

The unique capabilities of microfluidics directly address the most pressing challenges in developing effective wearable and implantable diagnostics [16]. First and foremost, the system's ability to operate on exceptionally low sample volumes is crucial. This is a non‐negotiable requirement for non‐invasive monitoring, where the available biofluids, i.e., sweat from a pore, tears from the corner of an eye, or interstitial fluid drawn through the skin, are produced in minuscule quantities [17]. A traditional lab assay may require millilitres of blood, but a microfluidic sensor can perform a similar analysis using microliters or less of sweat [18]. This capability eliminates the need for bulky sample reservoirs, making frequent, even continuous sampling feasible without depleting the source or causing user discomfort. The patient burden is dramatically reduced, moving from the unpleasantness of a needle stick to the passive, often unfelt collection of a biofluid [19]. Second, microfluidics enables remarkably rapid analysis. The short distances molecules must travel to react with a sensor, combined with the highly efficient mass transport within microchannels, slashes the time required for a complete assay from hours in a central lab to mere seconds or minutes at the point‐of‐need [20]. This acceleration is critical for real‐time feedback, whether it's alerting a person with diabetes to a crashing glucose level or an athlete to dehydration. The speed turns data into actionable information that can immediately influence behavior or trigger a therapeutic response.

Furthermore, multiple microfluidic channels and sensing zones can be fabricated in parallel on a single device, allowing simultaneous measurement of multiple biomarkers from a single, tiny sample [21]. This multiplexed analysis moves diagnostics beyond a single data point toward a more holistic biomarker signature, providing a more comprehensive picture of health status. For instance, a single wearable patch could measure glucose, lactate, electrolytes, and cortisol levels from a small volume of sweat, providing simultaneous insights into metabolic activity, hydration, and stress [22]. Finally, this integrated microfluidic system can be directly coupled with a suite of miniaturised biosensors, transforming the biomarker's chemical information into a digital electronic signal that can be wirelessly transmitted to a smartphone or a cloud platform. It is this elegant synergy between microfluidics, which handles the biological sample, and biosensors that transduce the signal, that creates a functional, self‐contained diagnostic device. By mastering the flow of fluids at the smallest scales, microfluidics provides an essential platform for building intelligent, responsive integrated systems that promise to redefine our relationship with personal health management.

The evolution of wearable and implantable technologies finds its special purpose and justification in the transformative potential of real‐time and non‐invasive diagnostics [23]. The clinical value of continuous data collection is profound, enabling a movement toward predictive and personalised medicine. Real‐time data enables doctors to observe the direct physiological effects of insulin doses, revealing patterns and correlations previously obscured [24, 25]. Those detailed data allow the development of highly individualised treatment plans, gradually moving away from the one‐size‐fits‐all protocols of the past. This could encourage therapies that are finely tuned to the unique rhythm of an individual's physiology.

The integration of digital health and artificial intelligence with microfluidic systems represents a transformative convergence that extends beyond traditional biosensing. AI‐driven analytics have enhanced the ability to extract reliable information from complex, high‐noise trace biological samples, enabling the correction of environmental interference, compensation for sensor drift, and accurate reverse‐engineering of multiple biomarker concentrations from raw signals. Furthermore, deploying lightweight AI models on devices enables low‐latency, real‐time analysis and autonomous, closed‐loop control. Yet this integration introduces new translational challenges, which are algorithmic bias arising from training datasets that inadequately represent diverse populations, and the absence of standardized validation frameworks that can ensure equitable performance across demographic groups. However, clinical translation faces persistent barriers beyond algorithms: patient‐centric factors such as wearability and adherence, systemic challenges including Electronic Health Record integration and data interoperability, and technical hurdles such as biofouling, power constraints, manufacturing scalability, and long‐term device stability under dynamic physiological conditions.

This review articulates a central thesis that microfluidics is a pivotal technological catalyst, enabling a new generation of wearable and implantable systems for personalised health. Our objective is to develop a systematic and critical translational analysis framework aimed at transforming technological possibilities into clinical reality, with the core of addressing the multi‐level trust deficit of ‘data‐algorithm‐ecosystem’ for understanding how this synergy is transforming the conceptual foundation of healthcare from a reactive model to one that is continuous, adaptive, and personalised. As shown in Figure 1, we contend that microfluidic platforms uniquely enable the transition from theory to practice by miniaturising fluid handling and integrating complex chemical processes at scale. On the diagnostic side, this includes extracting and analysing biofluids such as sweat and tears for biomarkers of metabolism, stress, and hydration. For future development, the ultimate success of microfluidics‐enabled personalized medicine hinges not only on hardware innovation but, more critically, on confronting the intertwined challenges of data heterogeneity, algorithmic bias, and the absence of rigorous clinical validation frameworks. Given the inherent differences in biological body fluids across individuals and situations, how to avoid systematic bias in artificial intelligence models to provide safer, more reliable results is worth further exploration.

**FIGURE 1:**
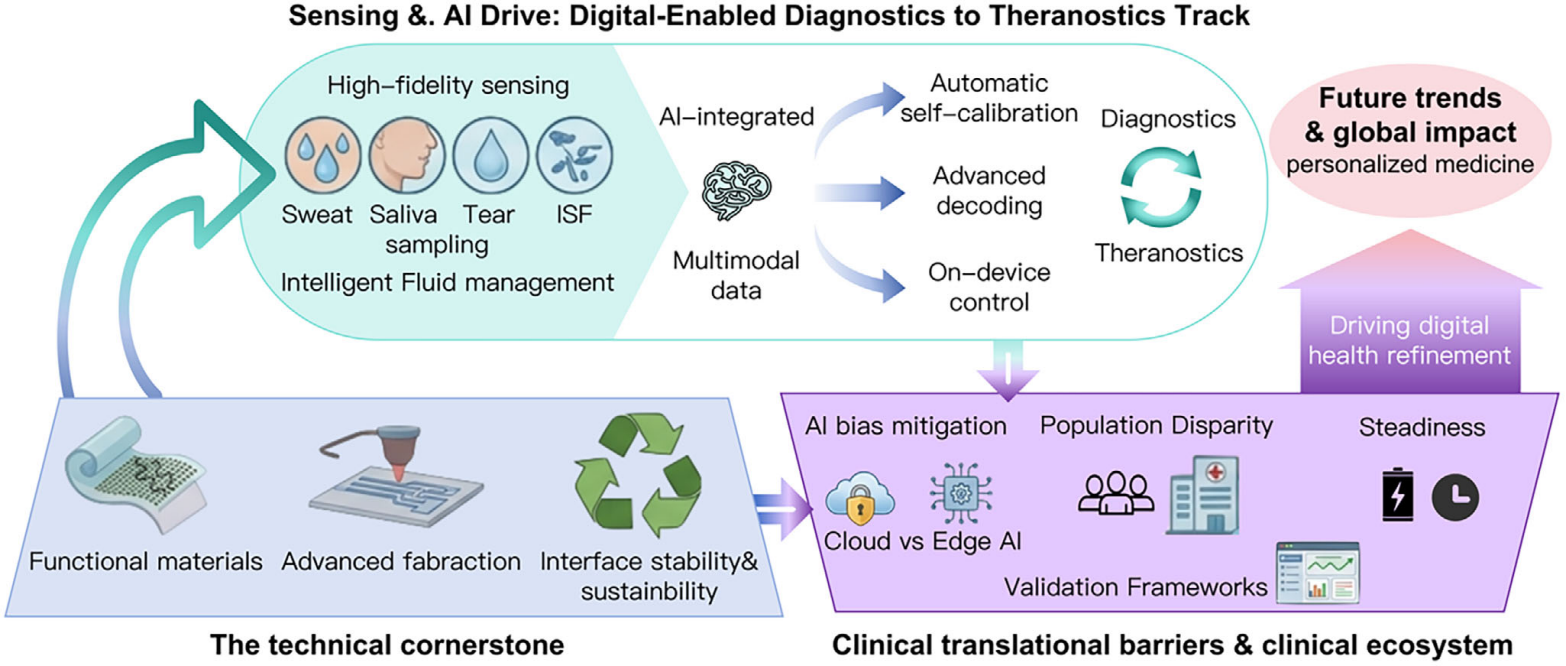
An overview of microfluidic wearable systems for clinical translation, covering material selection, device design, sensor integration, and AI‐driven approaches.

## 2 The Technical Cornerstone of Microfluidic Wearable Systems

### 2.1 Materials and System Design for Wearable and Implantable Integration

The transformation of microfluidic principles into functional health monitoring platforms demands sophisticated design strategies that address the unique challenges of human integration. Successful implementation requires a holistic approach that balances analytical performance with biological compatibility, power constraints, and user acceptability. A fully functional wearable or implantable device consists of a sensing system, a power system, and a communication system for processing, receiving, and transmitting signals [26]. These systems are designed to deliver intelligent management and services for the biohealth sector [27]. Polydimethylsiloxane (PDMS) has emerged as the predominant material for research prototypes due to its optical transparency, gas permeability, and established soft lithography protocols [28, 29]. These characteristics prove particularly valuable for wearable applications that require continuous contact with human skin and the monitoring of dissolved gases. Recently, advances in polymer science have led to the development of materials with enhanced mechanical properties and improved environmental stability. For implantable systems, material requirements become more stringent, necessitating materials that demonstrate chronic biocompatibility and minimal foreign‐body response. Parylene‐C coatings have emerged as a gold standard for implantable microdevices due to their unique combination of physicochemical properties, which could provide excellent barrier properties and biocompatibility [30, 31].

Wearable platforms must accommodate the body's dynamic biomechanics. Flexible and stretchable electronics enable reliable function under stretching, bending, and twisting. As shown in Figure 2A, epidermal electronics principles have been extended to microfluidic components and sensors integrated onto skin‐matched elastomers [32]. This strategy ensures conformal contact with curved skin surfaces and stable sensor–tissue coupling during movement. Electronics match the epidermis in thickness, mass, and stiffness, achieved via ultrathin, serpentine circuits fabricated on silicon wafers and transferred to PDMS membranes. Van der Waals lamination enables direct skin mounting. The resulting platform simultaneously measures strain, temperature, and electrophysiological signals, and serves as a foundational design. Recent advances, including serpentine interconnects, island–bridge layouts, and stress‐engineered geometries, improve stretchability and durability under repeated deformation [33]. System‐level integration places microfluidic channels on the same thin elastomer and links them to on‐board electronics for electrochemical detection (Figure 2B) [34]. yielding a lab‐grade, skin‐mounted sweat sensor for intense exercise. Multilayer stacking (Figure 2C) supports concurrent volumetric, colorimetric, and electrochemical sweat analysis, establishing a benchmark for 3D microfluidic–electronic integration.

**FIGURE 2:**
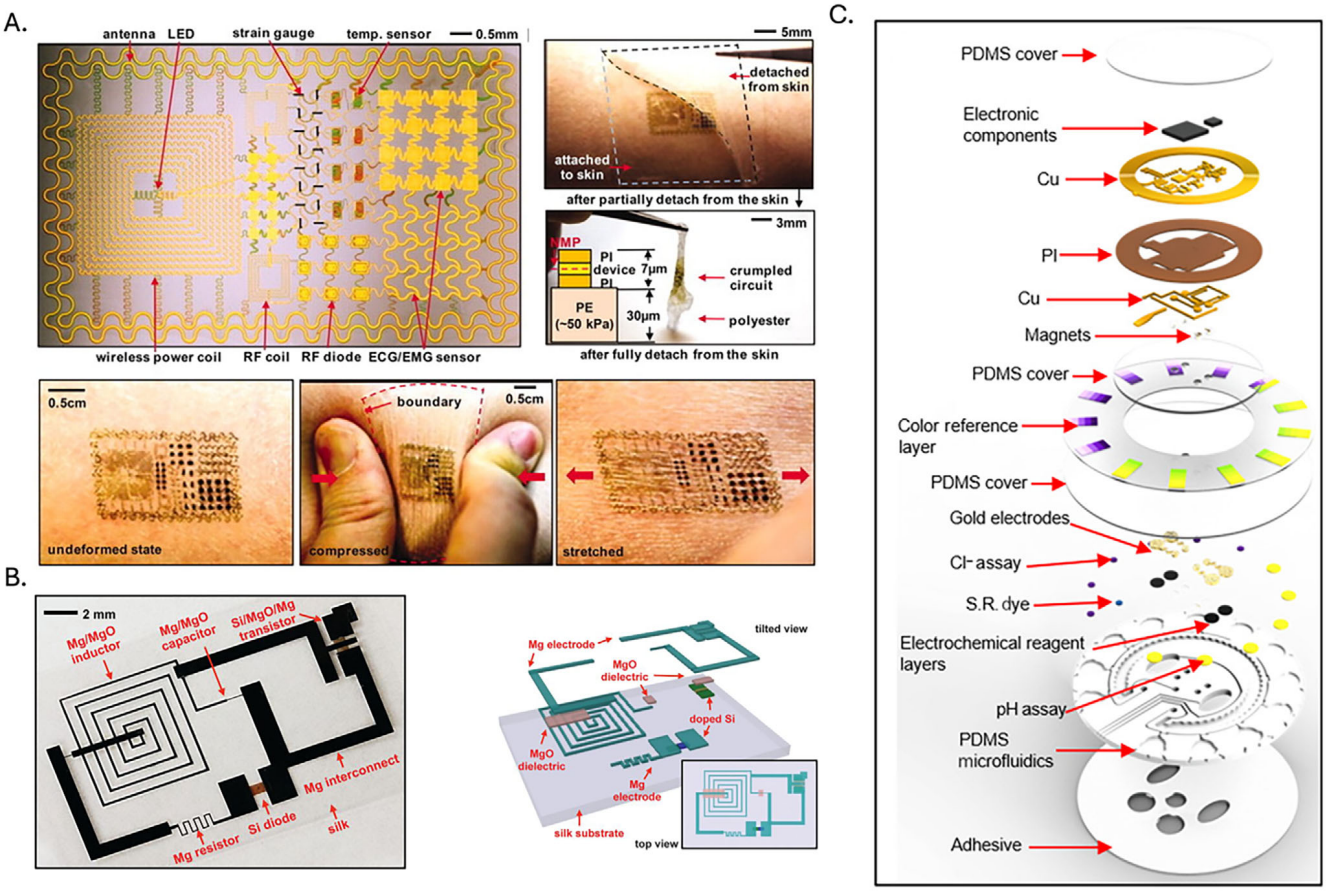
Design of conformal wearable devices. (A) Representative images of the demonstration platform mimicking the human epidermis for multifunctional electronics with physical properties. Reproduced with permission [32]. Copyright 2011, American Association for the Advancement of Science. (B) Schematic diagram of the transient electronics composed of its primary materials, device structures, and reaction mechanisms. Reproduced with permission [34]. Copyright 2012, American Association for the Advancement of Science. (C) Schematic diagram of the structure and components of the hybrid battery‐free microfluidic system. Reproduced with permission [33]. Copyright 2019, American Association for the Advancement of Science.

Accurate detection of specific biomarkers in complex biological fluids is a fundamental challenge and a primary objective of microfluidic wearable systems. Sweat, saliva, interstitial fluid, and blood could carry a different blend of proteins, salts, metabolites, and cells [35]. The same analyte can appear at different concentrations and at different time points across these matrices. Achieving this requires sophisticated detection pathways that combine biological recognition elements with precise signal transduction mechanisms.

Enzyme‐based electrochemical sensing is among the most established approaches for monitoring metabolic biomarkers, leveraging the specificity of biological catalysts that accelerate chemical reactions involving a narrow class of substrates. Wang et al. designed a skin‐interfaced patch called ‘NutriTrek’ that simultaneously monitors glucose and lactate levels in sweat during exercise using enzyme‐based electrochemical sensors, demonstrating the practical utility of this approach for real‐time metabolic tracking (Figure 3A) [36]. The glucose sensor utilizes the enzyme glucose oxidase, which catalyzes the oxidation of glucose. The generation of electron flow that produces an electrical current is directly proportional to the concentration of glucose present. Similarly, Zheng et al. demonstrated a wearable lactate sensor that incorporates lactate oxidase immobilised on a flexible electrode through epidermal methods, as illustrated in Figure 3B [37, 38]. During exercise, the enzyme catalyses the conversion of lactate and oxygen, producing hydrogen peroxide. The subsequent electrochemical oxidation of hydrogen peroxide at the electrode surface generated a current directly proportional to the lactate concentration in sweat. This allowed users to correlate sweat lactate levels with the intensity of physical exertion in real time, demonstrating the potential to optimise athletic training and monitor metabolic fatigue. These studies demonstrate the practical utility of enzyme‐based approaches for real‐time metabolic tracking during exercise. Ye et al. designed a wearable nanobiosensor to detect noninvasive female hormones, such as estrogen and progesterone, using aptamers as the primary biorecognition element (Figure 3C) [39]. A nanostructured electrode surface provides a high surface area for aptamer immobilisation, enabling a high signal‐to‐noise ratio and the detection of low physiological hormone concentrations in biofluids such as sweat or interstitial fluid [40, 41]. Mass spectrometry and immunoassays would be involved to obtain the data [42]. This work presents a practical approach to high‐sensitivity and continuous molecular monitoring that is both non‐invasive and user‐centric, thereby completing the cycle from diagnosis to therapy.

**FIGURE 3:**
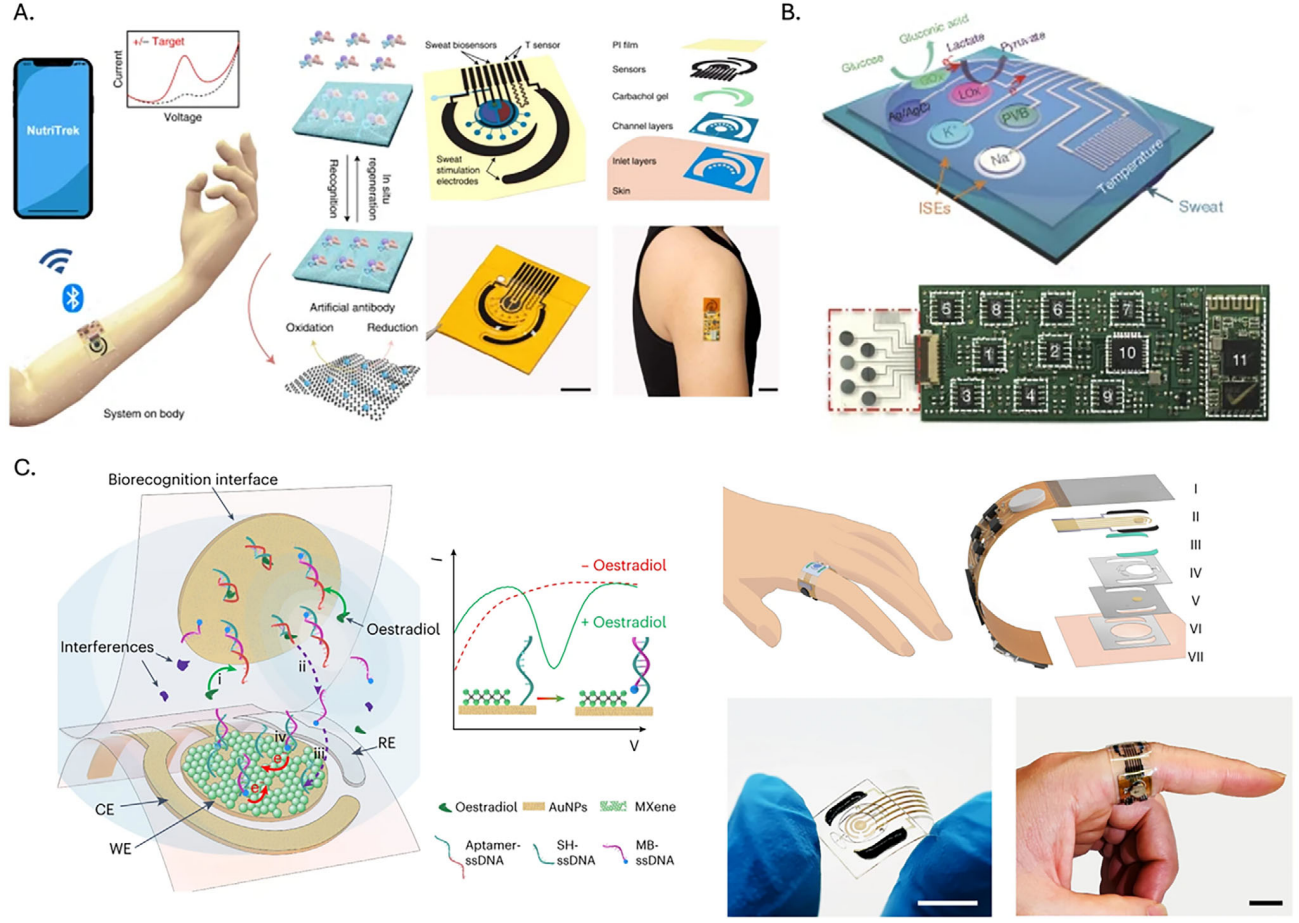
The microfluidic wearable system for detecting specific biomarkers in biological fluids. (A) Schematic and structure assembly of the microfluidic ‘NutriTrek’ device for sweat induction, sampling and biosensing. Reproduced with permission [36]. Copyright 2022, Springer Nature. (B) Schematic of the sensor array in the microfluidic platform, including glucose, lactate, sodium, potassium and temperature sensors, for multi‐channel analysis. Reproduced with permission [38]. Copyright 2016, Wiley‐VCH GmbH. (C) Schematic and structure design of the flexible and wireless microfluidic wearable nano‐biosensor for automatic sweat induction. Reproduced with permission [39]. Copyright 2024, Springer Nature.

A fundamental regulatory barrier for aptamer‐based sensing lies in the absence of standardized reference methods for validating measurements in complex biofluids. Gold‐standard techniques incompatible with wearable formats would delay clinical adoption and lead to regulatory uncertainty. The lack of established reference materials and standardized calibration protocols. The field critically requires collaborative efforts to establish reference methods and validation protocols specifically designed for continuous, non‐invasive monitoring platforms.

### 2.2 Fabrication Strategies and Material Optimization for Translational Microfluidics

The transformation of conceptual designs into functional wearable and implantable microfluidic devices relies critically on advanced fabrication techniques. Soft lithography, 3D printing, and laser micromachining encourage the creation of complex fluidic networks on substrates that can conform to dynamic biological tissues [43]. Each technique offers a unique balance of resolution, material compatibility, and scalability, making them suitable for different stages of development and application [44].

Soft lithography is a cornerstone technique for prototyping and producing PDMS‐based microfluidic devices [45]. A patterned silicon wafer serves as a master mold, typically created by photolithography. Its inherent flexibility enables integration with soft biological tissues, while its gas permeability is essential for applications such as cell culture or gas sensing in microchannels [46]. Unger et al. originally established multilayer soft lithography to construct fully elastomeric active microfluidic systems [47]. As shown in Figure 4A, Bossink et al. constitute a significant optimization of this paradigm to lower fabrication barriers, enlarge operational scales, and clarify application scenarios. The two are not replacements but rather represent the evolutionary trajectory of the same technological lineage. This recent advance reflected how the field has evolved from laboratory‐based invention toward application‐driven, translational technologies [48]. Xu et al. (Figure 4B) developed a method for integrating silicon‐based electronics with PDMS microfluidics using transfer printing. It separated each subsystem involving rigid semiconductor components and the biocompatible nature of PDMS fluidic networks. This hybrid strategy significantly expanded the capabilities of wearable microfluidic devices [49].

**FIGURE 4:**
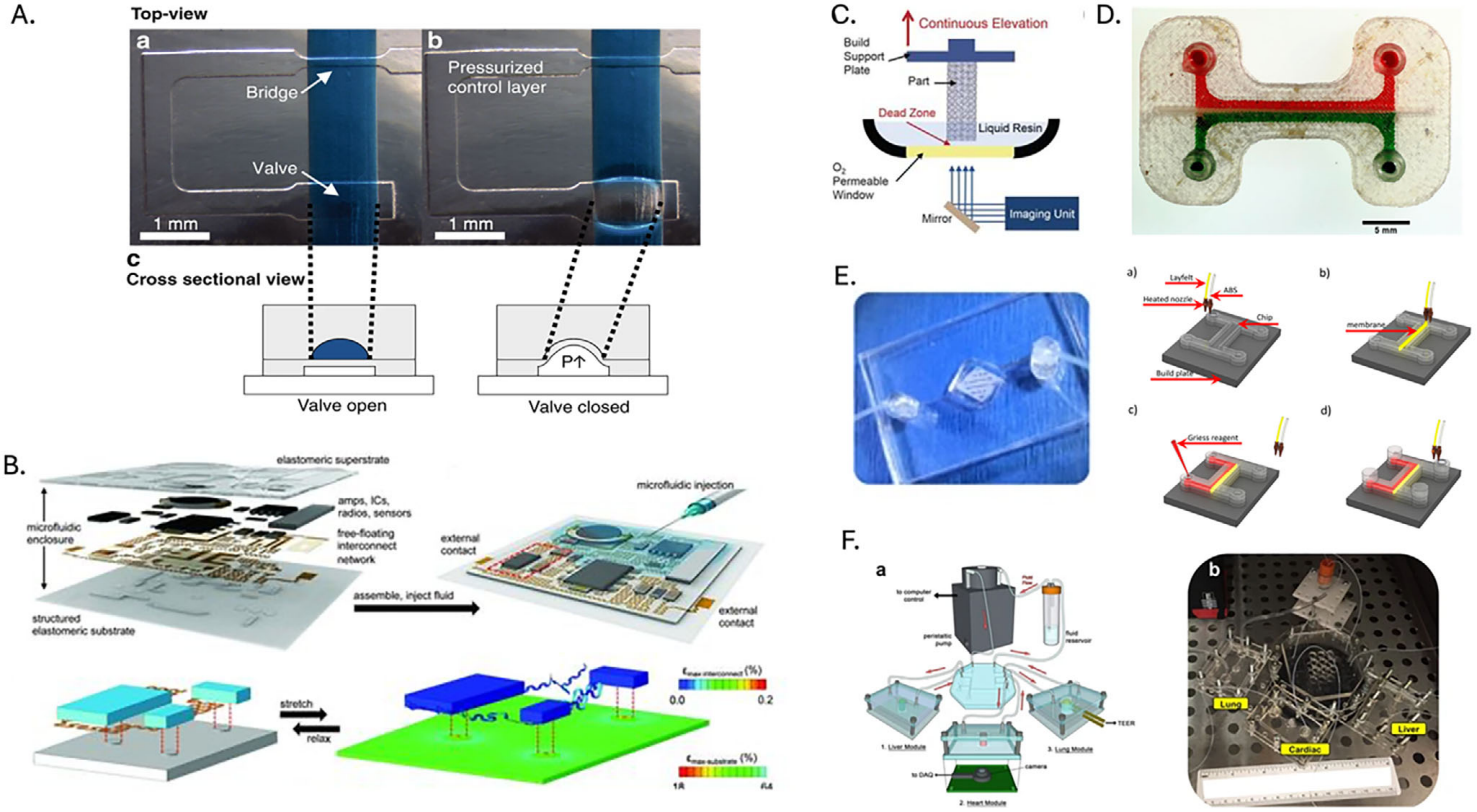
Different kinds of fabrication Techniques for microsystems. (A) Different views of cleanroom‐free fabricated macrovalves used in organ‐on‐chips. Reproduced with permission [49]. Copyright 2014, American Association for the Advancement of Science. (B) Schematic illustration of the key components of the system in different layers and 3D‐FEA results to show the flow associated with equal‐biaxial stretching of a small region of this platform. Reproduced with permission [50]. Copyright 2023, The Royal Society of Chemistry. (C) Schematic diagram of the simple architecture and operation of the CLIP printer. Reproduced with permission [52]. Copyright 2017, American Association for the Advancement of Science. (D) Representative images of a 3D printed chip integrated with a membrane. A 3D printing process has been demonstrated to produce a microfluidic chip. Reproduced with permission [53]. Copyright 2021, Multidisciplinary Digital Publishing Institute. (E). Representative images of how each tissue type was prepared in the separated system. Reproduced with permission [54]. Copyright 2017, Springer Nature. (F, G). Schematic drawing and photograph of the multi‐tissue OoC hardware system set up for maintenance of 3 3‐tissue model. Reproduced with permission [55]. Copyright 2021, Springer Nature.

Besides, 3D printing has emerged as a transformative digital fabrication technology [50]. Compared to traditional methods, 3D printing enables the direct fabrication of complex 3D channel networks and internal components in a single automated process. A high‐speed continuous liquid interface production method, as demonstrated by Tumbleston et al. (Figure 4C), has shown a fundamentally faster printing process that produces smooth microfluidic device components [51]. Li et al. have designed a microfluidic device with an integrated membrane printed using a FELIX 3D FDM printer, as illustrated in Figure 3D [52]. This work has demonstrated that the 3D printer can print specific structural components, functional semi‐permeable membranes, and pre‐loaded chemical reagents, thereby creating a ready‐to‐use analytical device directly from the printer. This approach relies on multi‐material voxel‐based printing, which defines the membrane as a distinct material region within the digital design. It could help to withstand repeated mechanical strain from body movement without failure. 3D printing techniques have the potential to develop in conjunction with organ‐on‐a‐chip (OoC) [53]. Skardal et al. have developed a three‐tissue OoC system composed of lung, liver, and heart, integrated with tissue organoids, as shown in Figure 4E–G [54]. The organs have been bioprinted into spherical organoids and then injected into a customised hydrogel chamber placed inside the microreactor devices. The drug responses depended on tissue‐tissue interactions, which could alter in vitro evaluations of both side effects and efficacy. Advances in 3D printing technologies continue to meet the unique requirements of wearable and implantable microfluidics. As these technical challenges are overcome, 3D printing is transitioning from a primarily prototyping tool to a viable manufacturing method for next‐generation personalised health monitoring systems.

The translational viability of 3D‐printed microfluidic devices warrants critical examination. The resolution limitations of most commercial 3D printers remain substantially coarser than the sub‐micrometer features achievable with photolithography, especially the complexity and density of fluidic networks [49]. The long‐term stability of 3D‐printed microstructures under physiological conditions has not been systematically investigated. The OoC application illustrates an instructive gap between technological promise and translational readiness. However, organoid viability only lasts for days to weeks, and the moderate‐to‐high complexity of cell culture maintenance renders clinical scale impossible. These limitations suggest that although 3D printing has transformed prototyping workflows and enabled innovative research platforms, its transition to usable manufacturing methods for clinical applications will require continuous advances in different aspects.

The successful integration of microfluidic systems with the human body requires a basic but important consideration of material choices. Traditional rigid electronics and brittle substrates prove inadequate for interfacing with soft, biologically active tissues, prompting the development of substrates for various purposes that form the physical foundation of next‐generation health monitors [55]. Advanced materials, such as polymers, parylene‐C, and Polylactic acid (PLA) [56], would exhibit enhanced mechanical effectiveness while maintaining harmonious contact with biological systems over extended periods.

Flexible, stretchable polymers are the cornerstone of wearable microfluidic devices 57. PDMS has been the material of choice for most research prototypes in recent years due to its inherent flexibility, optical transparency, and gas permeability. However, those materials are hydrophobic and have low tensile strength, which has driven the exploration of alternative elastomers. When PDMS is used in LOC applications, the PDMS surface affects molecular absorption, with the effect exacerbated at a favourable pH [58]. The inherent hydrophobicity of PDMS can be mitigated by plasma exposure, thereby enhancing protein absorption. Peng et al. (Figure 5A) have designed a microfluidic chip with a triple combination [59]. It combined PDMS for its elastomeric properties and ease of microchannel fabrication, glass for structural rigidity and optical clarity, and paper for passive wicking, eliminating the need for external pumps. The hybrid approaches improve PDMS hydrophilicity, extending shelf life or enabling prolonged use, but the lack of systematic data on long‐term stability hampers their transition from academic settings to clinical application.

**FIGURE 5:**
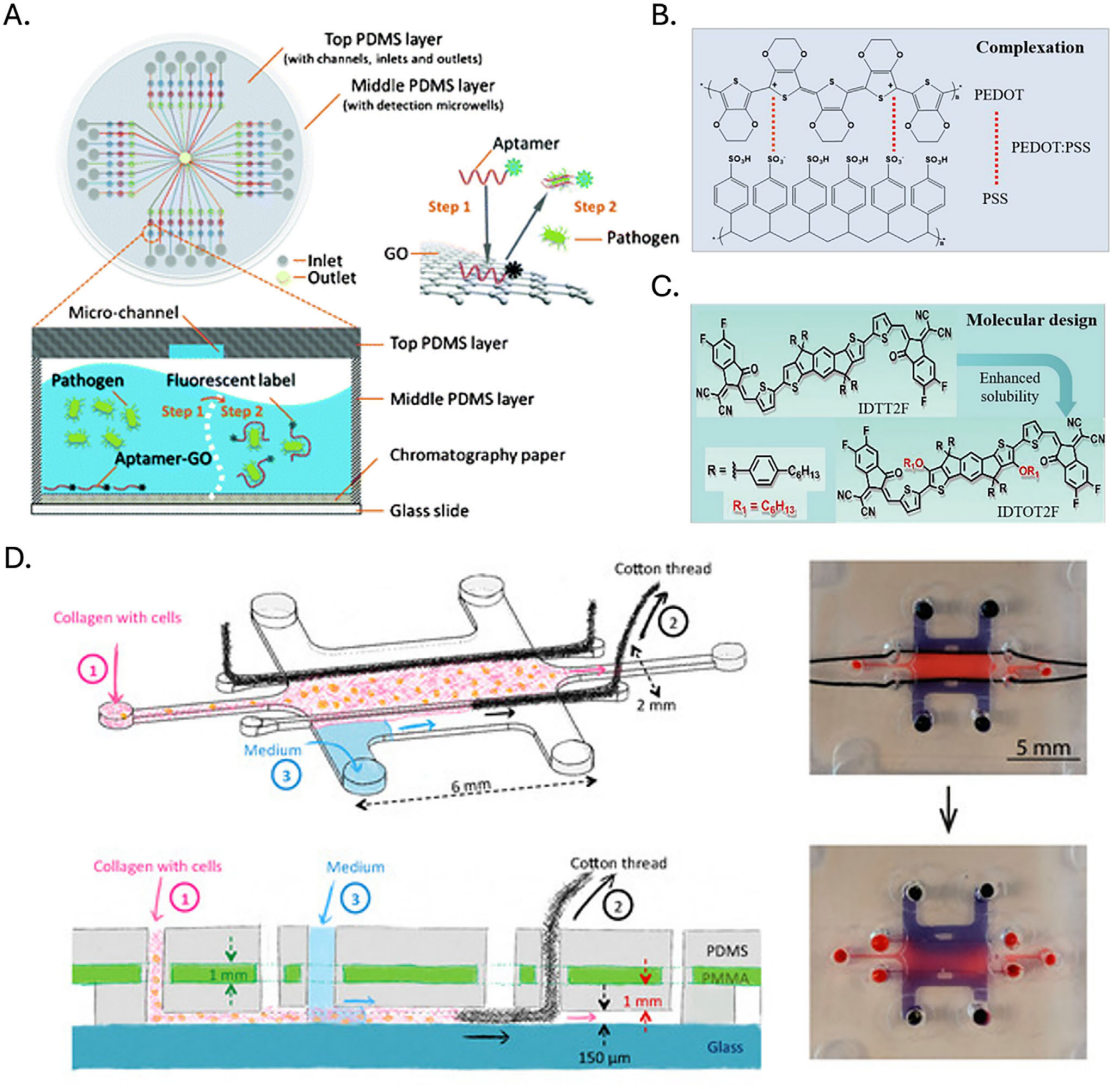
Different kinds of material optimisation for microsystems. (A) Schematic drawing of the PDMS/paper hybrid microfluidic system designed for single‐step multiplexed pathogen detection. Reproduced with permission [56]. Copyright 2023, Royal Society of Chemistry. (B) The structure of a polyelectrolyte complex. Reproduced with permission [59]. Copyright 2013, Elsevier. (C) The structure of the side chain group was introduced to improve the solubility of the molecules. Reproduced with permission [60]. Copyright 2020, American Chemical Society. (D) Schematic representation of the tumor‐on‐a‐chip device. Reproduced with permission [62]. Copyright 2018, MDPI.

Additionally, most organic polymers have lower conductivity than inorganic polymers [60]. The activity and stability of materials at human body temperature should be investigated when these materials are used in wearable and implanted devices, which could minimise the difficulty of the verification process and the operational cost of polymers for biosensor and device applications. The rapid progress in polymeric electronic devices is driving the development of new synthesis and polymerisation techniques. Developing a polymer with high potential for both electrical and mechanical properties could facilitate fabrication. They would form a polyelectrolyte complex by inducing specific agents to increase solubility (Figure 5B) [61]. The incorporation of suitable side‐chain groups into molecular structures can enhance the material's solubility in the solvent (Figure 5C) [62]. Polymethyl methacrylate (PMMA) is another typical polymer that does not deform under fluid pressure and has better biocompatibility than PDMS [63]. Valentina et al. designed a tumour‐on‐a‐chip model comprising a 3D tumour cell culture and an embedded PMMA sheet [64]. The combination of PDMS and microfluidic architectures for nutrient gradients and gas exchange is a common approach in biological studies. Both biomaterials played different roles in this microfluidic chip: PDMS for gas permeability and biocompatibility, and PMMA for structural rigidity.

### 2.3 Translational Considerations Based on Biocompatibility and Sustainability

The successful long‐term integration of implantable microfluidic devices presents an undeniable challenge that extends far beyond mere technical functionality, as concluded in Table 1 [65]. Long‐term function of implantation in tissue depends on how the device and the host adapt to each other over time. Device stability is critically important in the complex, dynamic environment of the human body.

**TABLE 1:** Comparative assessment of representative materials, fabrication techniques, and integrated systems presented to highlight the trade‐offs between device performance, biocompatibility, and immune response in microfluidic wearable and implantable technologies.

Reference	Material and method	Key advantages	Stability	Immune response
Bandodkar et al. [33]	3D multilayer microfluidic platform combining microfluidics, electronics, and battery‐free operation. Elastomeric substrates with serpentine interconnects.	Simultaneous volumetric, colorimetric, and electrochemical analyses. Battery‐free operation. Complete laboratory‐grade sweat analysis platform. It could withstand repeated stretching, bending, and twisting. Conformal skin contact.	Moderate. Multilayer integration can delaminate under strain. Electronics fatigue over extended cycles. The microchannel integrity under repeated mechanical stress. It has potential for leakage.	Low. For skin‐interfaced only and not implantable. Elastomeric materials are generally biocompatible. Minimal foreign body response. It is acceptable for short‐to‐medium term wearable applications.
Wang et al. [36]	Skin‐interfaced patch for sweat induction, sampling, biosensing. Disposable microfluidic design. Enzyme‐based electrochemical sensors, e.g., detecting glucose oxidase, lactate oxidase.	Simultaneous glucose and lactate monitoring in sweat during exercise. Real‐time metabolic tracking in a non‐invasive way. It provides immediate feedback. Wearable and user‐friendly design	Limited as enzyme‐based sensors have a typical functional lifetime of weeks. Glucose oxidase and lactate oxidase would denature over time. Sensor drift requires recalibration.	Low as skin‐interfaced only. It is non‐invasive and has no immune concerns. Minimal foreign body response, which is acceptable for short‐term wearable monitoring. Enzyme proteins may cause rare allergic reactions.
Ye et al. [39]	Flexible and wireless microfluidic wearable nano‐biosensor with aptamer‐based biorecognition elements. Nanostructured electrode surface. Automatic sweat induction with continuous molecular monitoring.	Non‐invasive female hormone detection, such as estrogen and progesterone. Its high sensitivity due to the nanostructured surface. It produces a high signal‐to‐noise ratio by detecting low physiological concentrations. Diagnostic to therapeutic cycle completion.	Good. Aptamers are more stable than antibodies, which can be engineered for enhanced stability. Less prone to denaturation. Nanostructures maintain performance over weeks to reduce sensor drift.	Low. Aptamers are nucleic acids (lower immunogenicity than proteins); Non‐invasive application. No immune concerns. Minimal foreign body response. No chronic implantation.
Bossink et al. [48]	Mechanical ‘macrovalves’ fabricated by multilayer soft lithography using micromilled direct molds. PDMS‐based pneumatic actuation with peristaltic pump and mixing device integration.	Enables organ‐on‐chip automation with flow rates matching physiological perfusion needs. Achieves complete fluid mixing within seconds for rapid reagent preparation. Provides programmable peristaltic pumping for long‐term cell culture.	Moderate. PDMS‐based valves exhibit fatigue over repeated cycling. Demonstrated biocompatibility for cell culture over multiple days under continuous perfusion; long‐term reliability beyond several days requires further investigation.	Low. PDMS is biocompatible. No implantation is involved. Suitable for in vitro organ‐on‐chip studies.
Xu et al. [49]	Transfer printing for integrating silicon‐based electronics with PDMS microfluidics. Hybrid strategy combining rigid semiconductor components with biocompatible PDMS fluidic networks. Multi‐layer system integration.	Expands wearable microfluidic device capabilities by combining electronics and fluidics in a single platform. Hybrid integration enables complex functionalities. Biocompatible fluidic networks with advanced electronics.	Good. Interface integrity between materials is critical. Silicon–PDMS adhesion must be optimized. Long‐term integrity depends on adhesive performance.	Complex. PDMS is biocompatible. Silicon is not in contact with tissue. Immune response is limited. Hybrid material compatibility requires careful assessment.
Li et al. [52]	FELIX 3D FDM printer with multi‐material voxel‐based printing. Integrated membrane fabrication with semi‐permeable membrane printing. Pre‐loaded chemical reagents for ready‐to‐use analytical devices.	Enables direct fabrication of functional structural components and semi‐permeable membranes. Pre‐loaded reagents create ready‐to‐use devices directly from the printer.	Moderate. PLA and PCL materials offer controlled degradation. Mechanical properties are stable for months. Printed layers may delaminate as layer adhesion is critical.	Good for approved materials; PLA is acceptable; concerns with additives and colorants; material certification is required; layer interfaces may affect immune response if implanted
Skardal et al. [54]	Three‐tissue organ‐on‐a‐chip (OoC) system integrated with tissue organoids. Bioprinted spherical organoids in customized hydrogel chambers within microreactor devices.	Enables tissue–tissue interactions for drug response evaluation. Provides both side effect and efficacy assessments. Reduces animal testing requirements.	Not applicable. Cells and media are changed regularly. Biofouling is only relevant to cell culture.	Immune response is not applicable. Used for in vitro drug testing only. Biocompatibility is assessed for cultured cells.
Rohland et al. [61]	Polyelectrolyte complexes with specific agents to increase solubility. Polymer synthesis and polymerization techniques are used for optimized electrical and mechanical properties.	Enables development of polymers with high potential for both electrical and mechanical properties. Facilitating fabrication and solubility. For flexible electronics applications	Good. Stable polyelectrolyte complexes with molecular engineering enhancing stability. Electrical properties are maintained to show the potential for long‐term function in physiological environments.	Low. Biocompatibility depends on molecular design. A stable water layer reduces immune recognition. It can be engineered for minimal foreign body response.
Liu et al. [62]	Side‐chain functionalization through introduction of suitable side‐chain groups. Molecular structure optimization to enhance solubility and processability.	Enhanced material solubility and improved solvent compatibility. Molecular engineering for processability. It enables easier device fabrication.	Good. Chemical stability is improved through molecular design. Consistent properties under electrical bias. Suitable for electronics integration.	Dependent on functional groups. Molecular design can minimize immunogenicity.
Valentina et al. [64]	Tumor‐on‐a‐chip model with 3D tumor cell culture and embedded PMMA sheet. PDMS and microfluidic architectures for nutrient gradients and gas exchange, combined with multiple biomaterials.	PDMS provides gas permeability and biocompatibility. PMMA provides structural rigidity, which enables nutrient gradients and gas exchange for biological studies. Combination approach leverages strengths of each material.	Moderate. PDMS–PMMA composite interfaces. Cell culture has a finite lifetime. Structural rigidity from PMMA. Gas exchange is maintained, but experimental durations are limited.	Not applicable. PDMS and PMMA are widely used. Biocompatibility is assessed for cultured cells. Cell attachment can be controlled.

Long‐term stability demands that materials withstand not only mechanical stresses but also a corrosive physiological setting [66]. Bettinger et al. explored the use of synthetic biodegradable polymers in medical applications, including conductive polymers and biodegradable elastomers, highlighting how molecular engineering can enhance operational longevity in aqueous ionic environments [67]. Numerous tests and experiments should be conducted to ensure their biocompatibility in both in vivo and in vitro settings. The necessity of selecting and designing materials not only for their initial properties in wearable implantation but also for their resilience over continuous yearly service within the human body.

Additionally, biofouling is one of the most significant challenges in developing long‐term implantation of microfluidic devices. The device surfaces could become targets for non‐specific protein adsorption, as plasma proteins would coat the foreign material. This could lead to the severe obstruction of fluidic channels, creating a diffusion barrier and resulting in an irreversible decline in measurement accuracy. Jiang et al. designed a new material that inherently resists initial protein adsorption [68]. A unique molecular structure enables the formation of an ultra‐dense, stable water layer via electrostatically induced hydration, which could be utilised as a potential coating for microfluidic devices. Wong et al. created Slippery Liquid‐Infused Porous Surfaces, a nanostructured surface within the device that can retain a lubricating fluid, resulting in an inert, ultralow‐fouling interface [69]. This could help adopt a microfluidic channel that demonstrates a biomimetic antifouling approach that is physically integrated into the device's structure.

On the other hand, any long‐term implant would face another significant hurdle: the foreign body response, a coordinated immune reaction that forms a collagenous capsule around the implant [70]. This phenomenon began with the rapid adsorption of a layer of host proteins onto the material's surface, creating a biological signature that the immune system can recognise. Finally, it would encourage the formation of an avascular and collagen‐rich fibrous capsule that completely walls off the implant from the surrounding tissue. It created a barrier that severed the critical connection between the sensor and the dynamic physiological environment. Veiseh et al. studied how tuning the material's chemical properties and physical geometry could dramatically reduce the extent of fibrous encapsulation, demonstrating that the foreign body response is not inevitable [71]. Besides the properties and characteristics of the materials used in microfluidic devices, their size and shape can also be potent regulators of the immune response. Implanting with sharp corners and edges consistently elicited a stronger foreign body response than implanting with smooth, rounded contours.

To address the long‐term challenges, scientists aim to enhance the biodegradability and sustainability of device materials. The pursuit of advanced medical implants necessitates a forward‐looking perspective on the ultimate fate of these devices within the body and their broader environmental impact [72]. The ideal implant for many temporary monitoring and therapeutic applications would perform its function with high accuracy over a period and then safely dissolve in the human body, minimising the long‐term burden on both the patient and the environment [73]. Irimia‐Vladu et al. suggested that synthetic polyesters, such as polylactic acid (PLA) and polycaprolactone (PCL), can be integrated into functional devices, offering a comprehensive alternative to persistent plastics like PDMS and paving the way for environmentally benign medical technologies [74]. It provided a biodegradable framework that met the demanding specifications of electronic devices. The challenges of achieving high performance without sacrificing environmental and biological responsibility have continued to improve in various ways.

The comparative analysis above reveals a consistent phenomenon across microfluidic wearable and implantable technologies: a high‐profile yet substantial gap between laboratory scale and clinical applications. Enzyme‐based electrochemical sensors for glucose and lactate monitoring have achieved a high level of clinical translation and are now widely adopted in diabetes management. However, most of the technologies still stop at the proof‐of‐concept stage. The absence of standardised testing protocols and limited data on long‐term stability at the clinical scale must be addressed before feasible clinical adoption spreads widely.

## 3 High‐Fidelity Wearable Microfluidic System for Personalized Health Applications

### 3.1 Wearable Microfluidic System for Diagnostics

Microfluidics plays a transformative role in personalised diagnostics by enabling the development of precise, efficient, and rapid testing methods tailored to individual health needs, consistent with modern personalised medicine care that centres on the patient [75]. One of the fundamental advantages of microfluidic technologies is their ability to create customised diagnostic tests based on a patient's unique health data, including genetic information, lifestyle factors, and medical history, allowing healthcare providers to design assays that target specific biomarkers relevant to a patient's condition, thereby fostering a more focused and effective approach to diagnosis [76]. Microfluidic technology enables personalized diagnosis by rapidly and sensitively analysing trace biofluids such as sweat and saliva, and interstitial fluid on wearable or implantable platforms, using only microliter‐ to nanoliter‐scale volumes [77, 78]. This capability has directly driven the transformation of point‐of‐care testing, shifting diagnosis from central laboratories to the patient's side and enabling immediate treatment adjustments based on individual results [79]. The role of artificial intelligence has enabled the system to go beyond measuring a single concentration and decode comprehensive feature spectra related to individual physiological states and disease progression from complex, multi‐source, time‐series data, achieving an intelligent leap from data collection to health insights. Further integration with digital health platforms enables real‐time aggregation and analysis of data, allowing diagnostic tests to be continuously optimised based on continuously collected health data and maintained in sync with patients' individual characteristics, thereby continuously enhancing the accuracy and clinical relevance of diagnoses [80].

#### 3.1.1 Intelligent Fluid Management and Multi‐Parameter Synchronous Analysis

The primary challenge in achieving personalised continuous monitoring lies in reliably and controllably collecting and analysing biological fluids with low secretion volumes and variable secretion rates. Active biofluid management is crucial for realizing wearable bioanalytical platforms that can autonomously provide frequent, real‐time, and accurate measurements of epidermally accessible biofluids such as sweat. Lin et al. have designed a programmable epidermal microfluidic valve system capable of sampling, routing, and partitioning biofluids for biomarker analysis [81]. The core of this system is a thermoresponsive hydrogel valve network controlled by individually addressable microheaters, supplemented by a pressure‐regulation mechanism to accommodate pressure build‐up when interfacing with sweat glands, as depicted in Figure 6A. This pressure‐regulating valve system features six compartments, each equipped with a distinct biochemical sensing interface integrated into its upstream channel, as illustrated in Figure 6B. This system achieves active guidance, on‐demand partitioning, and time‐resolved analysis of sweat samples through the synergy of a thermoresponsive hydrogel valve network and microheaters. The core innovation lies in intelligent fluid routing. By independently controlling the valves of each analytical unit, the system can direct sweat from specific time periods to the target sensors while protecting other sensors from contamination or cross‐interference, thereby enabling sequential measurements of multiple time points and markers from a single sample source. The corresponding sensing interfaces comprised an enzymatic layer to catalyze the oxidation of target molecules and generate hydrogen peroxide (H_2_O_2_) as a detectable byproduct, a permselective membrane poly‐m‐phenylenediamine) to reject interfering electroactive species, and a platinum electroanalysis layer to detect the generated H_2_O_2_, which is illustrated in Figure 6C. As shown in Figure 6D,E. For both sensors, linear relationships were observed between the measured current responses and the target analyte concentrations (R^2^ = 0.99). By integrating with a wireless flexible printed circuit board and enabling seamless bidirectional communication with consumer electronics, context‐related body biomarker data collection and display are realized. This design not only maximises the utilisation of the limited sample volume but also ensures data quality through active fluid control, a prerequisite for subsequent precise artificial intelligence (AI) analysis.

**FIGURE 6:**
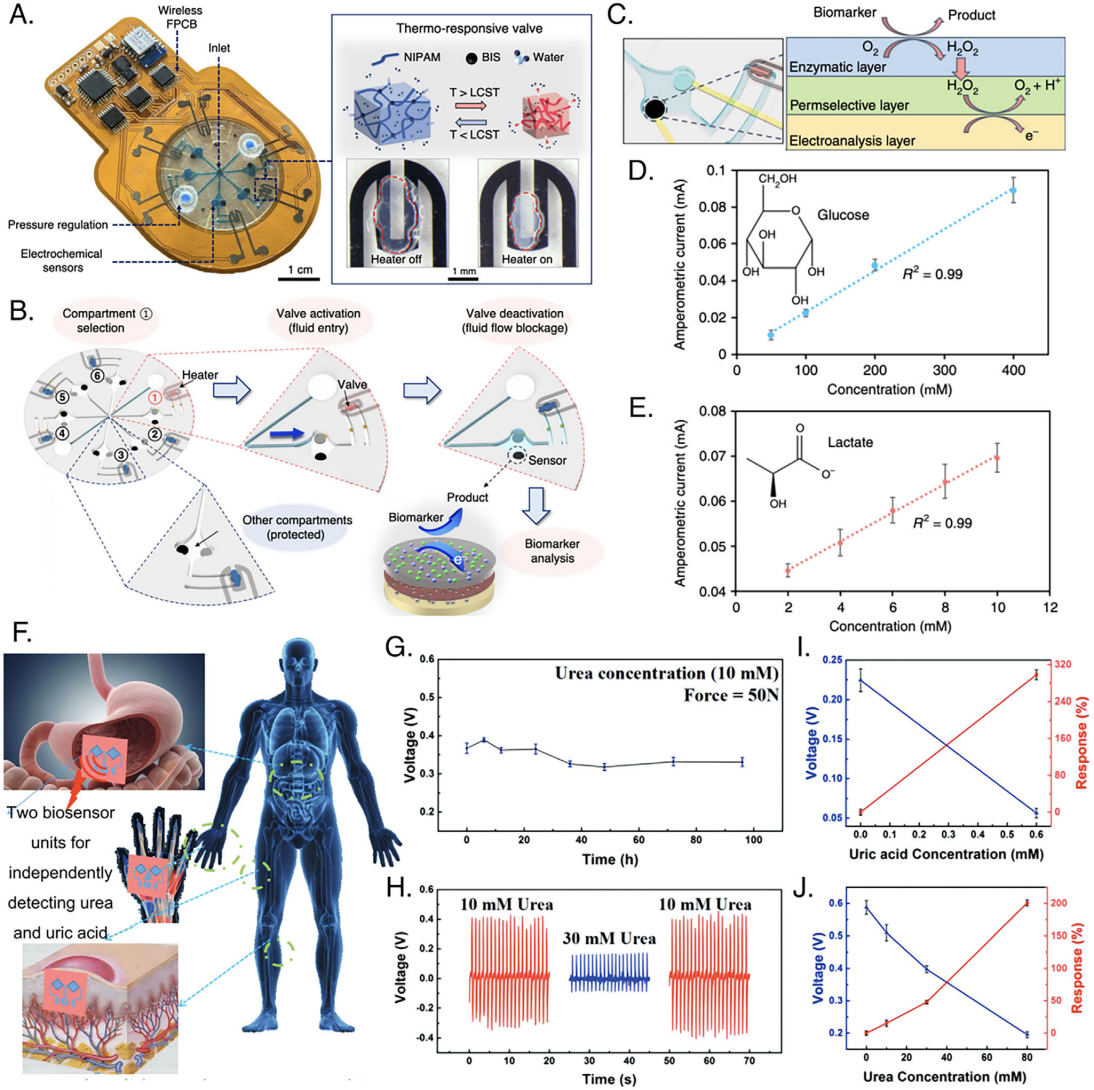
Customized microfluidic wearable sensors for personalized monitoring. (A) Illustration of a representative wearable bioanalytical platform, consisting of an integrated programmable microfluidic valving system interfacing a FPCB (right), and an illustration of PNIPAM hydrogel shrinkage/expansion in response to temperature change above/below its LCST (left). (B) A schematic operation example of the programmable microfluidic valving system, demonstrating biofluid routing, compartmentalization, and analysis in the selected compartment and sensor protection in the non‐selected compartments. (C) Reaction schematic of the developed sensor. Current response to target analytes for (D) glucose sensor, and (E) lactate sensor. Error bars, mean ± s.e. Reproduced with permission [81]. Copyright 2020, Springer Nature. (F) Experimental design: the e‐skin can harvest body‐movement energy and output body fluid information. (G) The lifetime of the device immersed in a 10 mM urea solution. (H) The reversibility of the device. (I) The response of the uricase‐modified unit. (J) The response of the urease‐modified unit. Reproduced with permission [82]. Copyright 2018, The Royal Society of Chemistry.

#### 3.1.2 Self‐Powered Implantable Sensing and In Situ Decoding of Body Fluids

To achieve truly long‐term, imperceptible, continuous monitoring, the device must address two major challenges: energy and biocompatibility. Yang et al. proposed a flexible, self‐powered implantable electronic skin (e‐skin) based on ZnO nanowire arrays, which are non‐toxic and have high energy conversion efficiency, for real‐time analysis of urea and uric acid in body fluids to diagnose kidney disease [82]. The e‐skin, shown in Figure 6F, converts mechanical energy from body movements into piezoelectric signals that reflect the concentrations of urea and uric acid, enabling specific detection via separate enzyme modification (urease/uricase). Each biosensor unit measures 5 mm × 5 mm, and the entire device covers approximately 1 cm^2^. Its stable output voltage has also demonstrated immunity to changes in applied force in actual use and can operate stably for more than 100 h, as shown in Figure 6G. The good reversibility is depicted in Figure 6H. This technology has enabled the relationship between voltage, urea/uric acid concentration, and in situ analysis response, as illustrated in Figure 6I,J, and offers a new method for self‐powered, real‐time diagnosis of chronic diseases through in situ fluid analysis.

#### 3.1.3 Digital Microfluidics and High‐Throughput Micro‐Sample Processing

Beyond customizing diagnostic tests, microfluidics enables rapid health assessment through innovative assays. These assays process small sample volumes quickly and efficiently, greatly shortening diagnostic turnaround times. High sensitivity is another key advantage, as microfluidic devices can detect low‐concentration biomarkers indicative of early‐stage diseases, particularly valuable in conditions like cancer, where early diagnosis is crucial for effective treatment. The microenvironment within microfluidic channels can be engineered to enhance detection signals, improving the chances of identifying critical biomarkers. The microenvironment within microfluidic channels can be optimised to amplify detection signals, thereby increasing the likelihood of identifying these critical biomarkers. Microfluidic technology reduces sample requirements by designing microscale fluid channels and droplet techniques. By generating discrete droplets at frequencies ranging from a few hertz to several thousand hertz, these independent microreactors can encapsulate biochemical reactants, cells, nanoparticles, nucleic acids, or other components and perform subsequent individual operations [83]. Electrochemical sensors are widely integrated into microfluidic systems for biological and chemical analysis due to their low reagent consumption, rapid detection, high signal‐to‐noise ratio, and minimal use of metallic materials [84]. Among these, digital microfluidics (DMF) is a specialized electrochemical technique that manipulates discrete droplets containing samples and reagents on a planar surface through amperometric analysis. Droplets are digitally controlled on a two‐dimensional array of uniform electrode units. Compared to conventional microchip‐based systems, DMF enhances reagent mixing uniformity, eliminates the need for micro‐pumps and valves, and simplifies device design and fabrication. Additionally, the independent control of each droplet enables dynamic reconfiguration, allowing operations at any array location and supporting parallel processing in a compact design during bioassays [85]. To address the severe matrix effects caused by blood cells and cell debris in whole blood sample testing. Dixon et al. introduced a novel DMF diagnostic platform integrated with a porous membrane, capable of directly collecting finger‐prick blood samples and separating plasma for multi‐step diagnostic tests [86]. The device comprises three core components: a plasma separation membrane (SM), a transport membrane (TM), and a fluid‐flow barrier, as illustrated in Figure 7A. The SM, derived from a commercial asymmetric membrane with larger pores on one side that gradually narrow, effectively traps hematocytes without causing lysis, allowing plasma to flow into the finer pores below, which is key to efficient plasma separation. The TM is a hydrophilic polyethersulfone membrane with low biomolecule binding (< 1%) and an average pore size of 5.0 µm, enabling rapid, consistent fluid flow. The fluid‐flow barrier consists of a wax plug (WP), which prevents uncontrolled reagent flow. The digital microfluidic device shown in Figure 7B features an inkjet‐printed bottom plate and an ITO‐PET top plate, forming a two‐plate configuration. In operation, SM captures red blood cells, TM delivers the filtered plasma to the electrode array, and WP controls plasma flow. Plasma separation, in‐line enzyme‐linked immunosorbent assay, and chemiluminescent detection were carried out in the extraction, assay, and detection zones, respectively. A 21‐step automated protocol was developed for the integrated separation and quantification of RV IgG in blood plasma, yielding a calibration curve spanning 0–30 IU/mL, as shown in Figure 7C. Linear regression analysis yielded an LOD of 1.9 IU/mL and an LOQ of 3.6 IU/mL, both below the WHO‐defined immunity threshold of 10 IU/mL for RV. Additionally, cyclic voltammetry (CV) has been employed for the quantitative analysis of samples. For instance, Srikanth et al. developed a simple, economical, and miniaturised chip‐based laboratory platform that simultaneously conducts bacterial culture and detection, using CV to monitor changes in bacterial concentration over time [87]. They integrated the microfluidic chamber with screen‐printed electrodes, as shown in Figure 7D, for the electrochemical detection of bacteria (Figure 7E). Additionally, they provided the required temperature for bacterial culture using an optimised laser‐induced graphene heater. Within the range of 2 × 10^^4^–1.1 × 10^^9^ CFU/mL, the device could quantify bacterial growth without altering the electrodes' biological properties. Fluorescence imaging was then used to confirm the activity of cultured bacteria in microfluidic devices. Micro‐microbial fuel cells were used to verify their metabolic activity, as shown in Figure 7F,G. This handheld and portable LOC platform was realized through 3D packaging and integrated with a portable potentiostat to achieve real‐time and field applications, which can be used for quantitative bacterial culture and has the advantages of simplicity, reliability, quantification, small sample size required, low cost, and low energy consumption.

**FIGURE 7:**
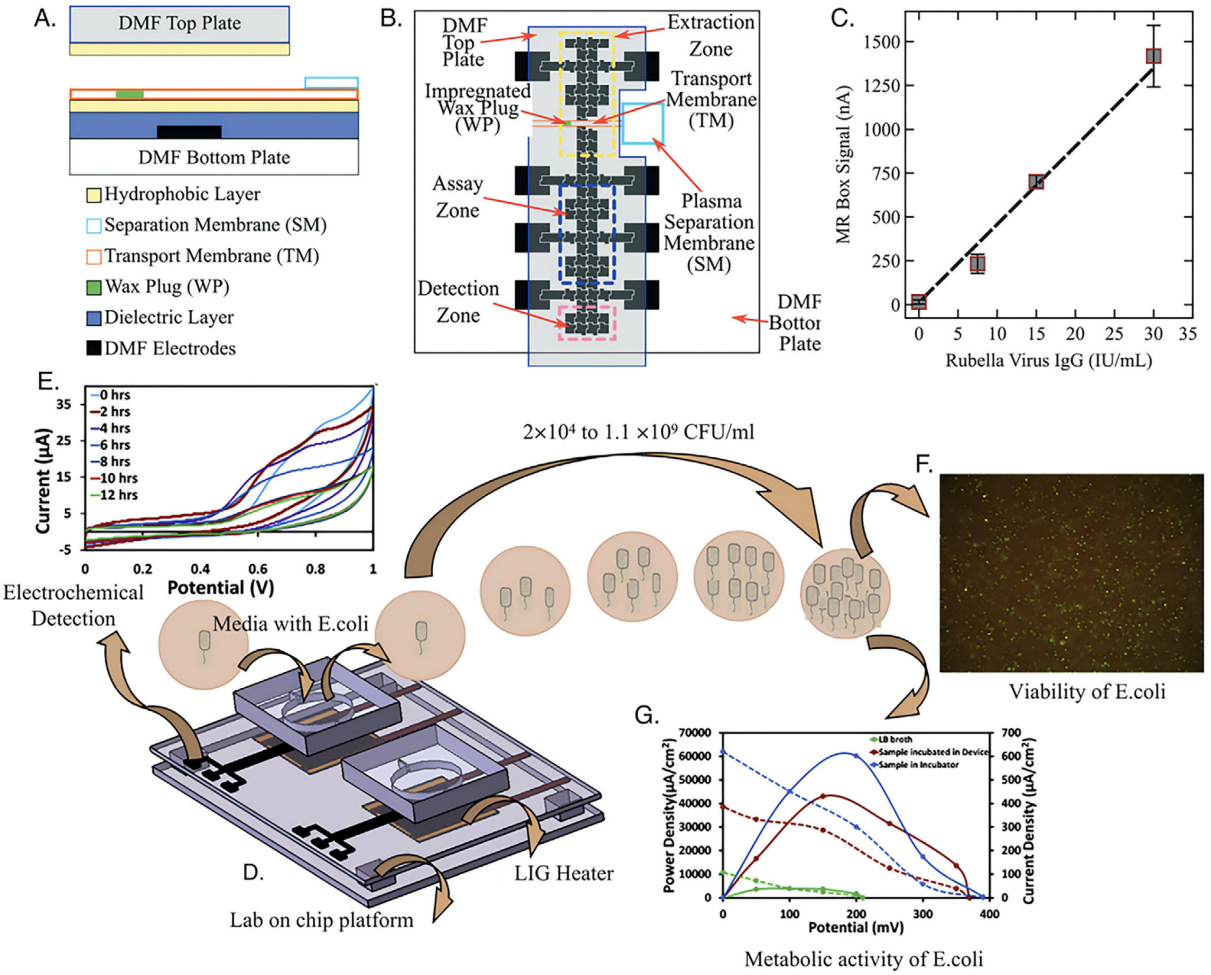
Wearable microfluidic sensors with high sensitivity for rapid analysis of small samples. (A) Cross‐section image of a complete two‐plate device bearing a DMF driving electrode (black) coated with dielectric (purple) and hydrophobic (yellow) layers. (B) Top‐view schematic of a complete two‐plate device with an inkjet‐printed bottom plate (black outline) and ITO‐PET top plate (blue outline), a plasma separation membrane (turquoise outline) for trapping red blood cells, transport membrane (orange outline) for delivery of filtered plasma into electrode array, and impregnated wax plug (green) to limit the flow of plasma in the TM. Plasma separation is performed in the extraction zone (yellow dashed box), in‐line ELISAs in the assay zone (blue dashed box), and chemiluminescent readout in the detection zone (pink dashed box). (C) Plot of chemiluminescence signal (markers) from RV‐IgG‐spiked blood samples as a function of concentration. Data were fit with a linear regression (dashed line). Reproduced with permission [86]. Copyright 2020, The Royal Society of Chemistry. (D) Microfluidics device integrated with screen‐printed electrodes and LIG heater. (E) Simultaneous electrochemical detection of bacteria. (F) Confirmation of viability through fluorescence imaging. (G) Validation of metabolic activity by employing the cultured bacteria for microbial fuel cell (polarization curves). Reproduced with permission [87]. Copyright2022, Elsevier.

#### 3.1.4 Multimodal Sensing and Data Fusion

Furthermore, microfluidic technology enables multiplexing, allowing multiple tests to be conducted simultaneously on a single sample. A single blood sample can be analysed for glucose levels, inflammatory markers, and other pertinent biomarkers, enabling a comprehensive assessment of the patient's condition [88, 89]. This capability enables rapid identification of multiple health conditions, providing a comprehensive overview of a patient's overall health status. Niu et al. have developed a fully elastic wearable electrochemical sweat detection system that integrates a sweat‐collection microfluidic chip, as shown in Figure 8A [90]. They proposed a unique bionic, tree‐like microfluidic chip structure that significantly improves the collection and drainage efficiency of fresh sweat, enabling electrochemical sensors to achieve real‐time detection, as shown in Figure 7B. This multi‐parameter electrochemical sensor for sweat can precisely and sensitively measure sodium ions, potassium ions, lactic acid, and glucose with high linearity, as depicted in Figure 8C–F. The electronic system is built on an elastic circuit board that perfectly conforms to the skin's wrinkles, enhancing wearing comfort and enabling multi‐channel data sampling, processing and wireless transmission. In addition, Zahed et al. provided a combination of biochemical and electrophysiological biosensing, as well as microfluidic channel‐integrated patch‐based wireless systems, utilising flexible materials for enhanced wearability, ease of operation, and real‐time continuous monitoring, as shown in Figure 8G [91]. The reduced graphene oxide‐based microfluidic glucose biosensor demonstrates a high sensitivity of 19.97 µA mM^−1^ cm^−2^ within the physiological range (10 µM–0.4 mM), along with excellent long‐term stability and mechanical flexibility under bending conditions. Integrated multimodal sensing of glucose and Electrocardiogram (ECG) signal, combined with real‐time correction for variations in sweat pH and temperature, enhances overall sensing accuracy, as shown in Figure 8H–J, which separately demonstrate the linearity of glucose, pH, and temperature. Additionally, laser‐burned hierarchical MXene‐polyvinylidene fluoride conductive carbon nanofiber dry ECG electrodes exhibit low skin contact impedance (40.5 kΩ cm^2^) and capture high‐fidelity electrophysiological signals with signal‐to‐noise ratios ranging from 23.4 to 32.8 dB, as shown in Figure 8K.

**FIGURE 8:**
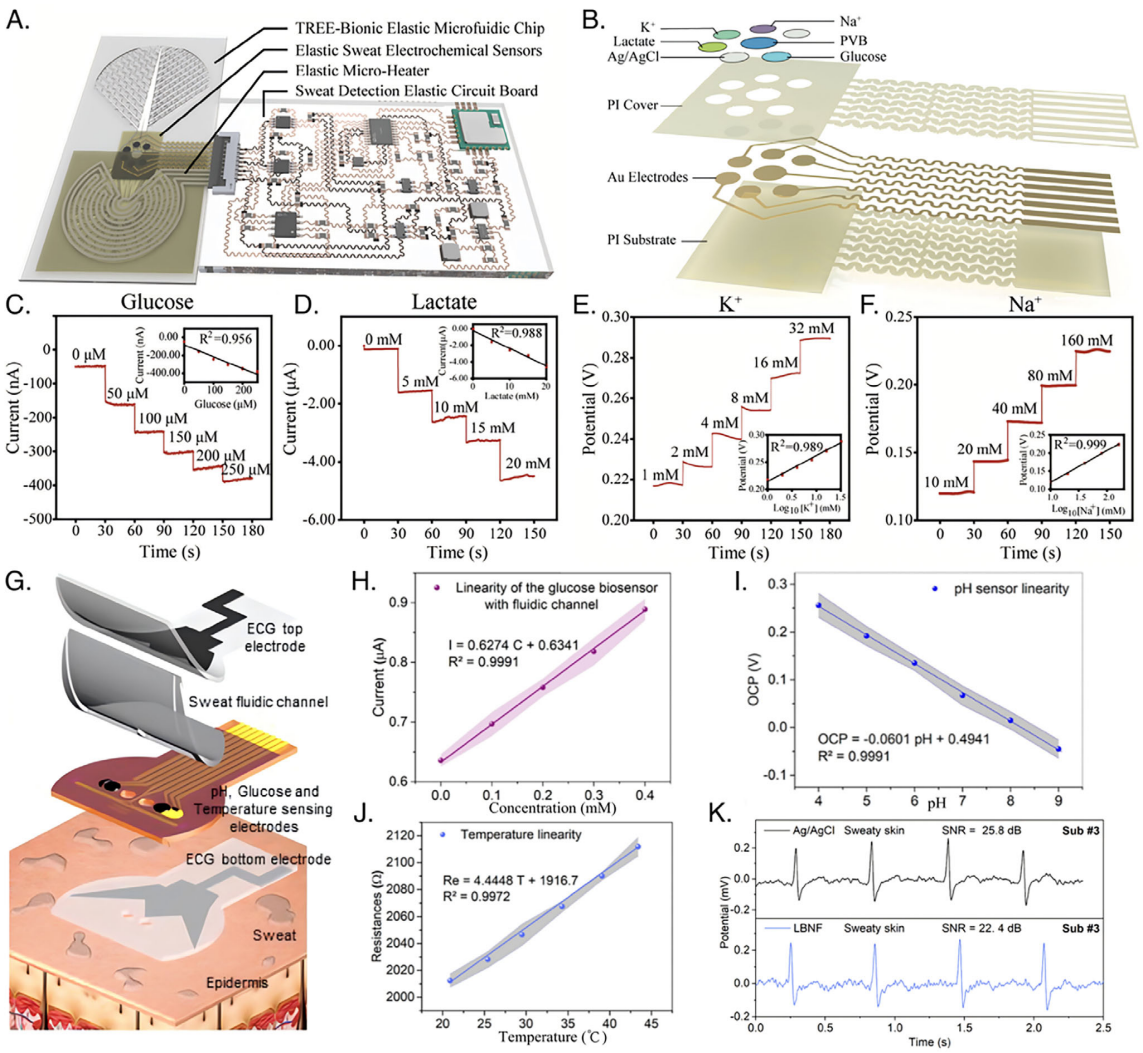
The multiplexed microfluidic wearable control sensor for monitoring can simultaneously conduct multiple tests on a single sample. (A) The 3D rendering of the Sweat system. The system comprises an ECB system and a microfluidics system component. The microfluidics system specifically includes a Tree‐bionic microfluidic chip, a sweat sensor, and a micro‐heater. (B) Schematic diagram of the structure and function of the electrochemical sensor. (C–F) The test results and linearity of the electrochemical sensors with standard sample solutions (1–32 mM KCl solution, 10–160 mM NaCl solution, 0–250 µm glucose solution, and 0–20 mM lactate solution) using an electrochemical workstation at 25°C. Reproduced with permission [90]. Copyright 2024, Wiley‐VCH GmbH. (G) Layer‐by‐layer illustration of the biopatch. (H) Linearity of the microfluidic‐integrated biosensor (the shaded region indicates the standard deviation of the biosensor after three repetitive tests). (I) Linearity of the pH sensor. (J) Linearity of the temperature sensor (the shaded region indicates the deviation of the three sensors). (K) ECG signal measured sequentially from the chest and right‐hand index finger of a subject utilizing Ag/AgCl electrodes (top) and dry LBNF electrodes (bottom) on sweaty skin. Reproduced with permission [91]. Copyright 2023, American Chemical Society.

#### 3.1.5 Rapid and Micro‐Scale Detection for Infectious Diseases

For infectious diseases, rapid microfluidic assays enable timely pathogen identification, a crucial step in making informed treatment decisions. For instance, such platforms can be designed to detect viral RNA or bacterial DNA in various specimens, rapidly facilitating the diagnosis of conditions such as COVID‐19, influenza, or sepsis [92, 93]. This capability not only streamlines the diagnostic process but also enhances healthcare providers' ability to make informed and personalised treatment decisions. Kong et al. developed a wearable microfluidic device that combines recombinase polymerase amplification (RPA) with body heat to enable rapid, straightforward amplification of HIV‐1 DNA, as shown in Figure 9A [94]. The flexible chip, featuring a single 50 µL fluidic chamber containing RPA reagents and HIV‐1 DNA, was immobilised by a wristband. The compatible temperature range for performing the RPA‐based assay was provided by heat transfer from the human body. After incubation, the chip was removed for fluorescence detection on cellphones, with HIV‐1 DNA detected at 10^2^–10^5^ copies/mL, exhibiting a log linearity of 0.98 within 24 min, as shown in Figure 9B. In addition, Gao et al. reported a flexible biosensing platform capable of multiplexed analysis of the wound microenvironment, including inflammation and infection status, at the point of care [95]. In Figure 9C, the wound dressing includes a perforated contact layer, a microfluidic exudate collector, an immunosensor, and a breathable barrier. The contact layer prevents direct contact between the immunosensor and the wound, thereby minimising disruption to the healing tissue. The barrier supports normal skin function by allowing oxygen to be absorbed and moisture to be released. The dressing's transparency enables real‐time visual monitoring of the wound. The immunosensor enables the detection of inflammatory mediators (tumour necrosis factor‐α, interleukin‐6 (IL‐6), IL‐8, and transforming growth factor‐β1), microbial load (Staphylococcus aureus), and physicochemical parameters (temperature and pH), as illustrated in Figure 9D. The sensor array is also integrated with a microfluidic wound exudate collector and flexible electronic components for wireless data transmission and smartphone‐based readout. In vivo multiplex monitoring was demonstrated in a murine wound model (Figure 9E), and clinical validation was performed using wound exudate from patients with venous leg ulcers (Figure 9F). This technology holds promise for enabling timely, personalised wound management, thereby enhancing outcomes in chronic wound healing.

**FIGURE 9:**
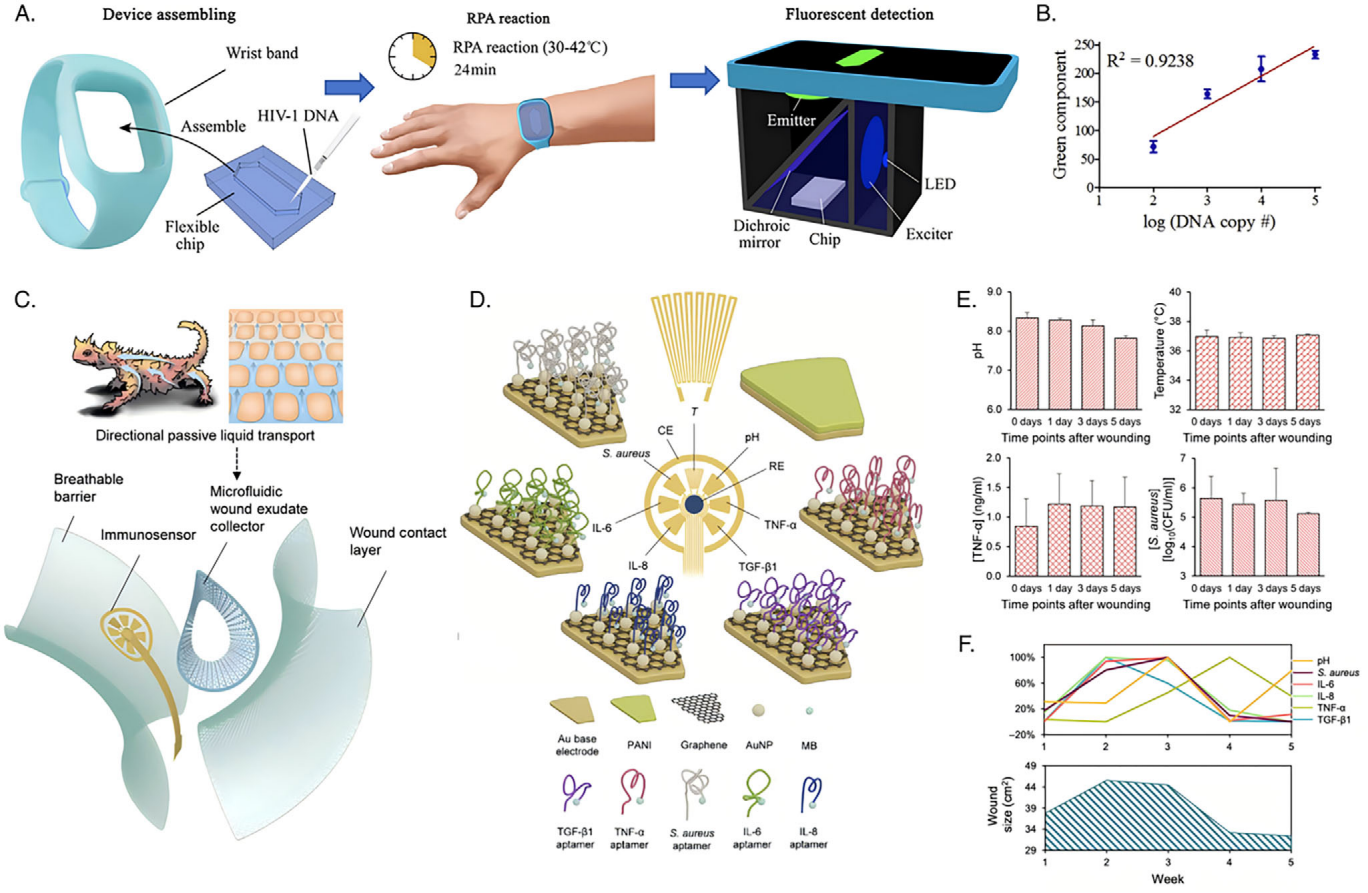
Microfluidic Wearables for Pathogen Detection. (A) Schematic of a single‐channel, PDMS‐based flexible chip, a flexible chip containing HIV‐1 DNA and RPA reagents assembled in a wristband, and then RPA was processed in the flexible chip via human body heat for 24 min to generate fluorescence signals. (B) Based on the CT values at 24 min, standard curves were constructed. Reproduced with permission [94]. Copyright 2019, American Chemical Society. (C) Envisioned biomarker analytical dressing constituting a perforated wound contact layer, a microfluidic wound exudate collector, an immunosensor, and a breathable barrier. The microfluidic collector was inspired by the skin of the Texas horned lizard, allowing a predetermined flow toward the lizard's snout and thereby defying gravity. (D) Schematic of the immunosensor for detection of TNF‐α, IL‐6, IL‐8, TGF‐β1, S. aureus, pH, and temperature. PANI, polyaniline; MB, methylene blue; RE, reference electrode; CE, counter electrode. (E) In situ assessment of pH, temperature, mouse TNF‐α, and S. aureus by the immunosensor. (F) Weekly assessment of pH, S. aureus, IL‐6, IL‐8, TNF‐α, and TGF‐β1 by the immunosensor for one patient. The axes represent independent scales for each quantified parameter, ranging from 0% (lowest) to 100% (highest). Weekly changes in wound size are shown, along with the biomarker assessment. Reproduced with permission [95]. Copyright 2021, American Chemical Society.

The microfluidic implementation of personalized diagnosis has evolved from the detection of single biomarkers to the intelligent management of trace and multi‐source biological fluids, simultaneous multi‐parameter analysis, and multi‐modal data fusion. These technological advancements have laid the foundation for generating high‐quality, high‐dimensional, individualised health datasets, representing a data supply‐side revolution for subsequent AI‐driven, in‐depth analysis.

### 3.2 Wearable Microfluidic System for Closed‐Loop Theranostics

The closed‐loop diagnosis and treatment system represents one of the ultimate goals of personalized medicine: based on real‐time monitoring data, it automatically triggers and adjusts treatment interventions to form a self‐adaptive health management closed loop. Microfluidic technology serves as a precise actuator in this process, responsible for storing, measuring, mixing, and delivering drugs in a controlled manner. At the same time, AI acts as the intelligent decision‐making centre, interpreting physiological signals, determining the timing of intervention, and identifying the best drug administration strategy. The collaboration of the two enables the transition from monitoring‐analysis to analysis‐decision‐making‐execution in a closed loop [96].

#### 3.2.1 Central Nervous System Intervention: Multi‐Functional Integration and Precise Localization

Closed‐loop interventions on complex organs such as the brain require devices with extremely high spatial accuracy, multifunctional integration, and minimal tissue damage. Shin et al. developed a multifunctional, multi‐shank MEMS neural probe, as shown in Figure 10A, that integrates optical stimulation, chemical delivery, and electrophysiological recording [97]. By embedding a dual‐purpose glass layer that serves as both a waveguide cladding and a microfluidic channel cover, the probe thickness is reduced to 40 µm, with a cross‐sectional area 6–8 times smaller than that of similar products, thereby significantly reducing the risk of tissue damage. On a single shank, a microfluidic mixer (µSHM) and an SU‐8 optical waveguide can be integrated, enabling real‐time mixing of two chemicals or dynamic concentration adjustment while supporting optogenetic stimulation. In Figure 10B, red‐ and blue‐dyed buffers from different inlets are sequentially injected into the 0.9% agarose gel at a specific flow rate. The infused volume from the probe is illustrated in Figure 10C. This highly integrated and minimally invasive design makes it possible to perform combined precise operations of ‘stimulation‐recording‐drug delivery’ in deep brain regions, providing a powerful tool for researching neural circuits and developing therapies for neurological diseases.

**FIGURE 10:**
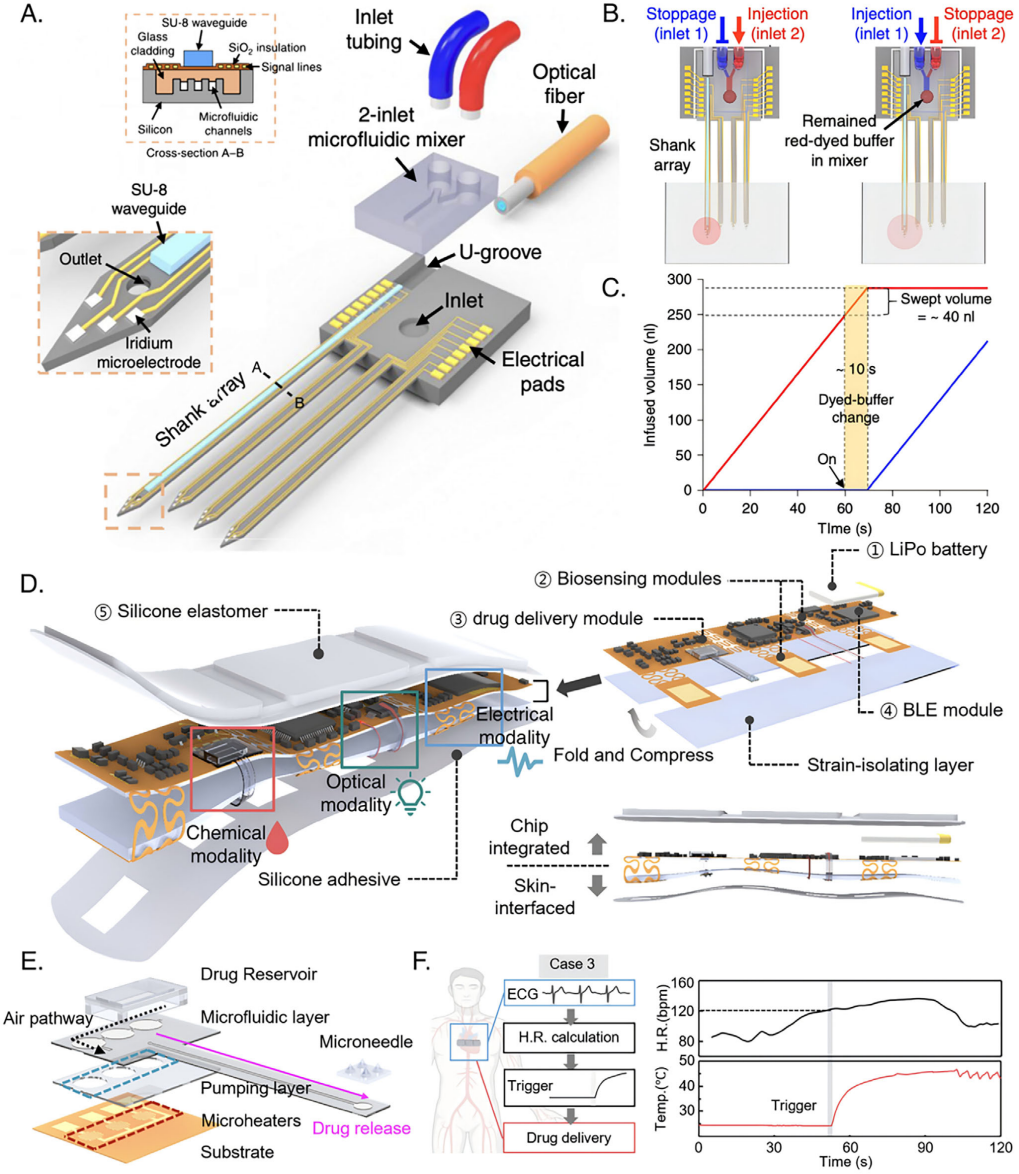
Closed‐loop microfluidic drug delivery systems. (A) Schematic diagram of the respective components before the device packaging, with an inset showing the probe tip. (B) Loaded red‐dyed buffer injection in inlet 2; A stoppage of red‐dyed buffer injection to the agarose gel and loaded blue‐dyed buffer injection in inlet 1 (right), and Continuous infusion of the blue‐dyed buffer into the agarose gel (left). (C) Plot of the infused volume of red‐ and blue‐dyed buffers from the probe to 0.9% agarose gel. Reproduced with permission [97]. Copyright 2019, Springer Nature. (D) Integrating and folding process of fMMD with multilayer components of chemical, optical, and electrical modalities. Each modality is denoted by red, green, and blue colored boxes, respectively. Each component is designed to be divided into rigid and soft layers. (E) Exploded view of the micropump‐based microfluidic system, composed of a soft drug delivery channel with a drug reservoir, a pumping membrane layer, and a microheater array for thermo‐pneumatically pumping actuation. (F) Schematic showing the principle of feedback operation of fMMD through ECG monitoring and drug delivery triggering, and real‐time measurement of HR and temperature of the microheater indicating initiation of drug delivery operation. Drug delivery is automatically triggered when the HR exceeds 120 bpm. Reproduced with permission [98]. Copyright 2025, Springer Nature.

#### 3.2.2 Transdermal Drug Delivery and Multimodal Physiological Feedback Closed Loop

For the daily management of chronic diseases such as cardiovascular diseases, wearable transdermal closed‐loop systems have greater practical prospects. Lee et al. introduced a vialess, multi‐modal skin patch that separates soft and strain‐robust regions using a folded structure [98]. The system incorporates both electrical and optical modalities for hemodynamic and cardiovascular monitoring, along with a force‐electrically driven micropump for precise drug delivery, as illustrated in Figure 10D. Each modality is demonstrated through specific functions: on‐demand drug delivery shows the microfluidic system's capability; flexible waveguide‐based PPG enables non‐invasive physiological monitoring; and ECG and motion tracking demonstrate the electrical sensing performance. For controlled transdermal drug delivery, the system comprises three key functional components: a micropump with a triggering mechanism to maintain a steady flow rate, a reservoir integrated with microfluidic channels to transport the drug from the storage unit to the skin, and a microneedle array for skin penetration. In Figure 10E, a micropump‐based microfluidic system utilises a thermopneumatic micropump driven by an array of microheaters, which generates a sustained pressure gradient through precise control of a general‐purpose input/output (GPIO) signal sequence, enabling force‐electrically assisted drug delivery. Figure 10F presents a schematic demonstrating real‐time feedback operation based on both electrical and chemical modalities. An external PC can collect ECG signals wirelessly and calculate HR in real time. When HR exceeds 120 bpm, the system initiates a feedback algorithm that sends a trigger packet to initiate drug delivery and start micropump operation. This case perfectly illustrates how to obtain physiological states through multi‐modal sensing, decode key features in real time via AI, and ultimately drive the microfluidic system to deliver trace amounts of drugs on demand, at scheduled times, and in precise quantities, thus forming a complete wearable therapeutic closed loop.

#### 3.2.3 Personalized Programmable Drug Delivery and Adaptive Strategies

The automated nature of these systems minimizes human error, reduces the risk of complications due to overdose or underdose, and enhances patient compliance by providing consistent therapeutic responses. In addition, based on the closed‐loop concept, feedback systems can utilise real‐time data to optimise drug administration plans. By integrating data from various sources, these systems create a dynamic treatment framework that tailors treatment to patients' specific needs. By adopting adaptive algorithms, feedback‐based systems can adjust drug dosages or administration rates in response to current physiological conditions, thereby precisely matching user needs. More complex clinical scenarios may require combining multiple drugs, timed administration, or adjustments to strategies based on composite biological signals. Babatain et al. reported a wearable, personalised drug‐delivery platform that integrates skin sensors, a processing unit, and a microfluidic drug‐delivery device [99]. The sensors monitor physiological signals, and after analysis by the processing unit, the heating element is triggered to control the expansion of the expandable layer, thereby regulating the drug flow rate. The structural design of the microchannel and the active heating‐triggering mechanism jointly influence drug release rate and dosing, enabling personalised treatment, as illustrated in Figure 11A.

**FIGURE 11:**
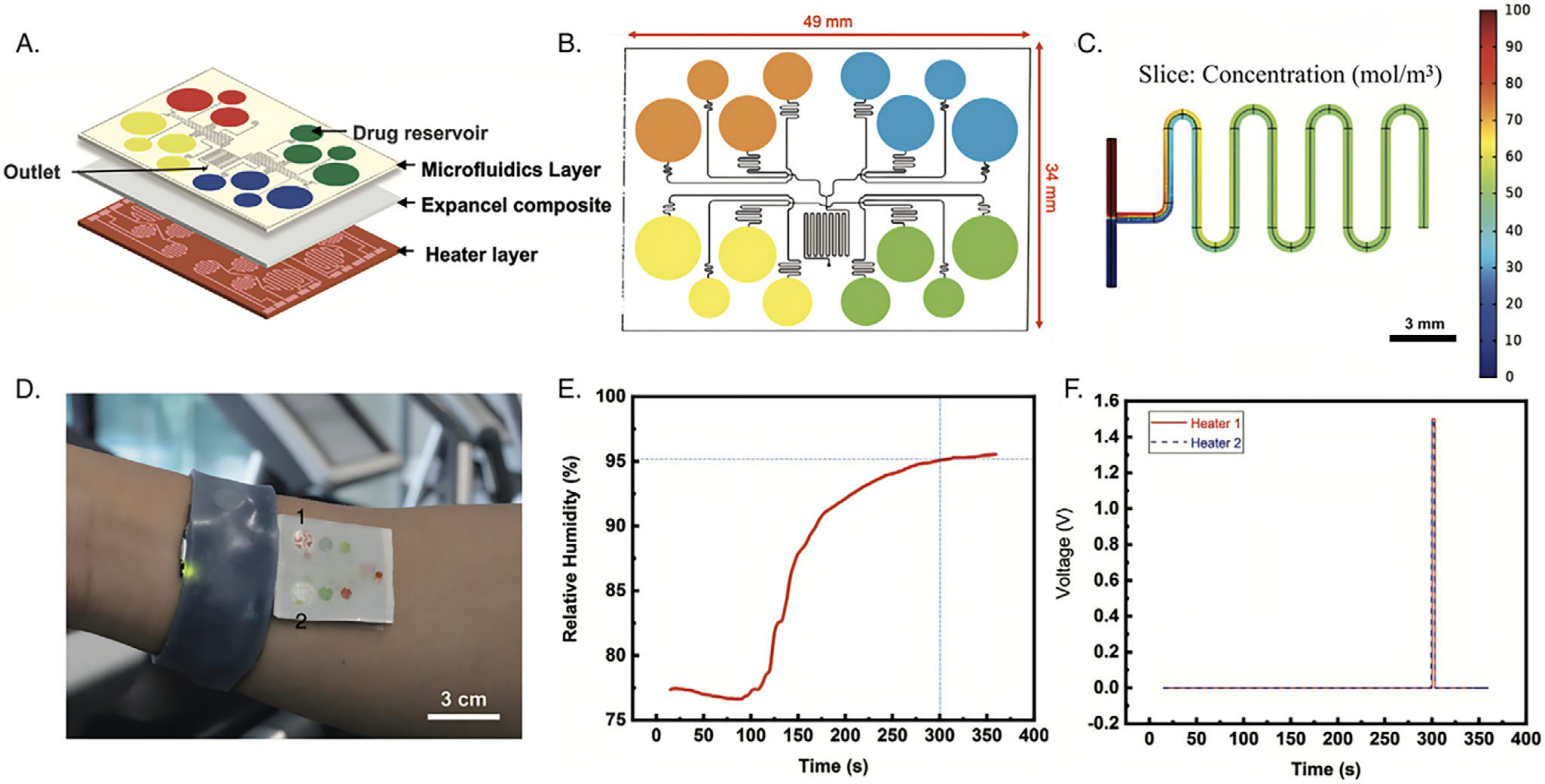
Feedback‐based wearable microfluidics platform. (A) Schematic illustration showing an exploded view of the device. (B) Design of the drug reservoir layer containing a network of microfluidic channels and mixers. (C) Mixing simulation results of a circular‐shaped channel. (D) The platform after actuation. (E) Closed‐loop feedback experiment. (F) Triggering the device after detecting physical fatigue from wearable sensors. Reproduced with permission [99]. Copyright 2020, WILEY‐VCH Verlag GmbH.

The design features 16 half‐ellipsoid‐shaped reservoirs with four capacities: 295, 225, 165, and 115 µL, as shown in Figure 11B. Once the heating element located beneath the expandable polymer is triggered, the temperature rises to approximately 80°C, causing the Expancel microspheres to expand sevenfold in volume. This irreversible expansion squeezes the PDMS microchannel, pushing the drug from the reservoir through the microneedles and into the skin. Moreover, the release rate can be controlled by adjusting the heating intensity (temperature and duration), and the multi‐reservoir design allows simultaneous or time‐separated release of different drugs. Serpentine‐semicircular‐based passive mixers in the microchannel achieve multi‐drug mixing through diffusion, as shown in Figure 11C. Drug flow is regulated by triggering the heating element in response to sensor data. The expansion of the polymer layer controls the release rate. The microchannel structure and heating control together determine the dosage and timing, enabling customized treatment. In the physical fatigue monitoring test in Figure 11D–F, once the preset threshold of 95% relative humidity is reached, the two heaters are immediately activated. As a result, the liquid is sprayed from the two preset accumulators, 1 and 2, and mixes before reaching the outlet. This study demonstrates the potential to define and reconfigure the functions of microfluidic pathways using AI algorithms, enabling a single hardware platform to execute highly customised and complex drug‐delivery schemes via different instructions and providing a hardware foundation for achieving dynamic, individualised treatment.

To bridge the theoretical mechanisms with translational applications, Table 2 provides a panoramic benchmark of representative wearable microfluidic systems, categorized strictly into diagnostic biosensors and closed‐loop theranostic platforms.

**TABLE 2:** Representative wearable and implantable microfluidic systems.

Reference	Biofluids	Analytes	Sensing methods	Detection range and LOD	Response time	Validation model and sample size	Substrate and fabrication	Limiting factors/Challenges
Passive capillary and evaporation‐driven microfluidic systems								
Yang et al. [100]	Eccrine sweat	Sweat rate, total loss, pH, Glucose, Lactate, Cl^−^	Colorimetric optical density (Chrono‐sampling/RGB color vector)	Rate: 0.1–5.0 µL/min Glucose: 0.05–0.5 mM Lactate: 5–30 mM	Real‐time flow readout; ∼5 min (color colorimetry)	Human cycling trials (indoor/outdoor athletics), N = 12	3D microfluidic network with capillary‐bursting valves; multilayer PDMS soft lithography	Colorimetric irreversibility prevents tracking downward concentration kinetics.
Mo et al. [101]	Natural epidermal sweat	β‐Hydroxybutyrate (β‐HB, Ketone body)	Fluorescence emission intensity (Thiourea donor lock‐and‐key hydrogen bonding)	10–200 µM LOD: 2.45 µM	∼2 min	Human fasting/ketogenic diet assessment, N = 6	BHI fluorescent probe in paper‐based reservoirs on flexible PDMS chip	Photobleaching of organic fluorophores restricts continuous multi‐day interrogation.
Ploner et al. [102]	Continuous sweat flow	Tumor Necrosis Factor‐alpha (TNF‐α)	Square wave voltammetry (SWV) peak current (Aptamer ratiometric strategy)	1–100 pg/mL LOD: 0.46 pg/mL	∼10 min (Affinity incubation lag)	In vitro simulated continuous sweat flow mimic	MB‐labeled aptamers on gold microelectrodes embedded in laser‐patterned PET	Reversible regeneration of aptamer sites remains challenging, leading to sensor saturation.
Li et al. [103]	Stimulated or Natural sweat	Creatinine, Urea, & Mg^2+^	Colorimetric RGB absorbance (Bioenzyme‐free H _2_MoO_5_/Cu^2+^ ternary catalyst)	Cre: 10–200 µM (LOD: 1.83 µM) Urea: 2–30 mM (LOD: 0.17 mM)	∼5 min	Human exercise trial & simulated artificial sweat testing, N = 3	Capillary bursting valves & reservoirs via laser‐cut PDMS soft lithography	Colorimetric irreversibility prevents tracking concentration drops; high ambient light interference.
Shin et al. [104]	Autonomously induced sweat	Glucose & Lactate	Amperometric current (Enzymatic oxidation calibration)	Glucose: 10–800 µM (LOD: 2.1 µM) Lactate: 2–30 mM (LOD: 0.16 mM)	∼20 s (electrode response); 10–20 min (fluidic renewal lag)	Multi‐day continuous human routine monitoring (sedentary lifestyle, > 60 h), N = 4	Hierarchically graded channels with Janus membranes on PET/PDMS	Localized carbachol iontophoresis triggers skin irritation during multi‐day usage.
Tu et al. [105]	Iontophoresis‐extracted sweat	C‐reactive protein (CRP), pH, Na^+^, Temp	Square wave voltammetry (SWV) (CRP antibody); Potentiometric voltage (Na^+^/pH)	CRP: 1 pM‐100 nM (LOD: 0.17 pM) pH: 4.0‐8.0	∼5 min (Antibody‐antigen electrochemical incubation)	Clinical cohort study (COPD, heart failure, and active infections vs. healthy), N = 36	Graphene array functionalized with gold nanoparticles & anti‐CRP on flexible PI/PDMS	Massive interpersonal/intrapersonal ionic strength variations perturb antibody binding.
Wearable microfluidic systems for closed‐loop theranostics								
Ge et al. [106]	Wound exudate	Temperature & Humidity (Controlled drug delivery)	Resistive resistance (Temperature); Capacitive impedance (Moisture monitoring)	Temp: 20°C–50°C Humidity: 0%–100% RH	Physical sensors: < 5 s; On‐demand heat drug release: ∼2 min	In vivo chronic wound healing evaluation using infected Sprague‐Dawley (SD) rat models, N = 5 rats/group	Liquid metal (LM) heating circuits & hydrogels inside asymmetric wetting porous fabric patch	Chemical degradation and oxidation of the liquid metal lines under prolonged exposure to chronic wound exudate.

Based on the above research, more intelligent sensors can adjust the dosing plan in real time in response to monitoring indicators, thereby enabling a long‐term, dynamic, closed‐loop monitoring system for drug delivery. The integration of microfluidic technology into personalized health applications has paved the way for more effective diagnosis and treatment. At the same time, monitoring accuracy, material selection, and drug transport control are key factors that determine the device's effectiveness and should be adjusted appropriately to meet specific monitoring goals. By enabling customised detection and adaptive drug‐delivery systems, microfluidic technology enhances the precision of medical interventions tailored to individual patient needs.

## 4 The Integration of Digital Health and Artificial Intelligence

The ultimate value of microfluidic wearable and implantable systems lies in transforming continuously collected microphysiological data into actionable clinical insights. This clinical translation engine relies on a seamless, bidirectional synergy between microfluidic hardware and algorithmic intelligence, executing a highly coordinated data‐to‐decision pipeline. However, successfully bridging the gap between raw biophysical readouts and precise, personalized medical interventions requires addressing a series of multiscale, system‐level bottlenecks throughout the entire signal lifecycle. To transition these platforms from passive empirical monitors to active therapeutic agents, the development of these technologies must move toward mathematically rigorous algorithmic frameworks that execute a continuous loop of automatic calibration, microscale decoding, and adaptive feedback, all while adhering to strict safety and regulatory boundaries.

### 4.1 Automatic Calibration for Accuracy Improvement

AI has fundamentally enhanced the ability to extract reliable information from complex and high‐noise trace biological sample data. Through machine learning (ML) and deep learning (DL) algorithms, AI can correct environmental interference, compensate for sensor drift, and directly and accurately reverse‐engineer the concentrations of multiple biomarkers from raw sensor signals [107]. Wang et al. introduced an AI‐assisted wearable microfluidic colourimetric sensor system (AI‐WMCS) for the rapid, non‐invasive, and simultaneous detection of key biomarkers in human tears, including vitamin C, H^+^ (pH), Ca^2+^, and proteins, as demonstrated in Figure 12A [108]. To enhance accuracy, by collecting a large number of color data of each concentration gradient marker under different color temperature and pH conditions, a well‐trained multichannel convolutional recurrent neural network CNN‐GRU is used to correct errors in the interpreted concentration data caused by varying pH and color temperature in different measurements, and a 3D dataset as one of the characteristic signals for the training of the 3D‐CNN‐GRU model, obtaining the calibration curves of the other three biomarkers (vitamin C, Ca^2+^, and proteins) under different pH, thus achieving pH corrections for the concentration prediction of vitamin C, Ca^2+^, and proteins, as shown in Figure 12B. The determination coefficients (R^2^) for the 1D‐CNN‐GRU in predicting pH values and for the 3D‐CNN‐GRU in predicting the other three biomarkers were 0.998 and 0.994, respectively. Figure 12C–F shows the correlations between the concentrations of four target biomarkers measured by the AI‐WMCS microsystem and the actual concentration values, with high Pearson's correlation coefficients. In addition, based on users' physiological data and historical records, AI can generate personalised health advice to help users improve their lifestyles, such as dietary adjustments and exercise plans [109, 110]. This fully demonstrates how AI, as a core analytical engine, ensures the accuracy and robustness of results from trace samples in complex matrices, thereby upgrading traditional colourimetric methods.

**FIGURE 12:**
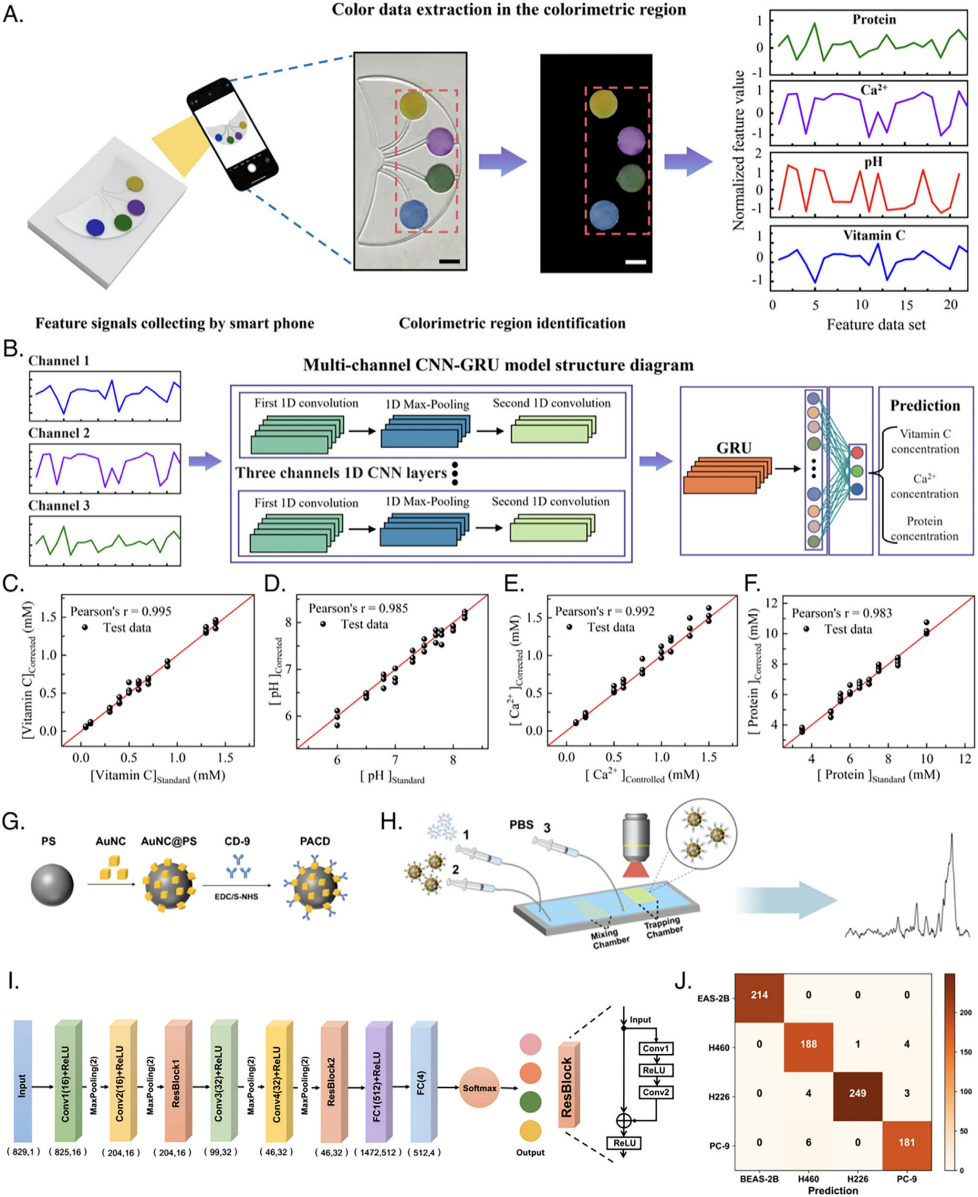
AI‐assisted biomarker detection systems. (A) Illustration of the color data acquisition through a smartphone and extraction of the feature datasets from the four colorimetric regions in the captured photo. Scale bars: 2 cm. (B) Structure diagram of multichannel CNN‐GRU model with three feature signals of length 21 as input and three biomarker concentrations as output. (C–F) Comparison of pH and color temperature‐corrected concentrations of (C) vitamin (C, D) pH, (E) Ca^2+^, and (F) proteins with the actual concentration values and the corresponding Pearson's coefficient. Reproduced with permission [108]. Copyright 2024, Springer Nature. (G) The synthesis of the exosome capture probe PACD. (H) Microfluidic‐SERS Chip for exosome capture and SERS detection. (I) Architecture of the deep learning model and the ResBlock. (J) The confusion matrix of deep learning‐driven exosome classification. Reproduced with permission [111]. Copyright 2025, American Chemical Society.

### 4.2 Advanced Decoding: Molecular Profiling From Trace Nanoscale Targets

For early cancer screening, although tissue biopsy is the gold standard, it is invasive and costly and provides limited information about the tumour and its molecular profile. AI can also provide more accurate diagnostic results. The target biomarkers, such as exosomes, have extremely low concentrations and high heterogeneity, placing greater demands on the analytical techniques’ sensitivity and specificity. This requires AI not only to handle concentration information but also to decode high‐dimensional molecular fingerprint spectra. Chen et al. reported a deep learning‐based microfluidic chip combined with surface‐enhanced Raman scattering (SERS) readout for characterising differences among exosomes, facilitating the early diagnosis and molecular typing of non‐small cell lung cancer (NSCLC), thereby demonstrating this advanced decoding capability [111]. The integrated system comprises an exosome capture and enrichment processing unit utilising polystyrene microspheres, gold nanocubes (AuNCs), and an anti‐CD‐9 antibody, as illustrated in Figure 12G.

Additionally, it features an optical sensing unit, shown in Figure 12H, for capturing PACD and detecting the SERS signals of these exosomes. As shown in Figure 12I, the network consists of an input layer, four convolutional layers, four pooling layers, two residual blocks, two fully connected layers, and an output layer. The spectral data range is 300–1800 cm^−1^, with 829 1D data points as input. A ReLU activation function is applied after each convolutional layer to enhance the model's expressive power. The system achieved a maximum capture efficiency of up to 85%. It distinguished between three NSCLC cell lines and regular cell lines, achieving an overall accuracy of up to 97.88%, as shown in Figure 12J. It is not merely a measurement of concentration but also an intelligent identification and typing of molecular identity information carried by nanoscale targets separated from macroscopic samples, opening new avenues for non‐invasive liquid biopsy and precise molecular diagnosis.

### 4.3 On‐Device AI and Autonomous Closed‐Loop Decision‐Making

The highest level of integration is to deploy lightweight AI models on the device, enabling low‐latency, localised, real‐time analysis and autonomous, closed‐loop control. This is crucial for conditions such as epilepsy and severe hypoglycemia that require immediate intervention. For instance, Ouyang et al. have proposed a wireless system that can be implanted in the body without physical wires or batteries to record and regulate neural circuits [112]. The system can be implanted under the skin of the back of freely moving small animals to autonomously record electroencephalograms, electromyograms, and body temperatures, and achieve closed‐loop neural regulation through optogenetics and pharmacology. Among them, programmable closed‐loop pharmacology, enabled by electroencephalogram feedback, can effectively inhibit epileptic seizures. The data analysis process is divided into two steps in Figure 13A. First, a deep learning model is trained on a workstation equipped with a GPU accelerator to generate a compressed executable file, which is then uploaded to the BLE system‐on‐chip, thereby forming an optimised data pipeline that can perform data preprocessing, deep learning inference, and closed‐loop control with limited computing resources. The demonstration presented here employs a convolutional neural network (CNN), which achieves excellent balanced performance in epileptic seizure detection, offering higher accuracy and moderate computational complexity compared to artificial neural networks (ANNs) and long short‐term memory (LSTM) models. The time series data is transformed into spectrograms through short‐time Fourier Transform (STFT), each with a shape of 35 × 31. The deviation caused by the signal amplitude is eliminated by dividing the signal amplitude by the total power within each time interval, thereby forming the training features. The CNN model contains only 12 002 parameters and 300 000 multiply‐add (MAC) operations. It is composed in sequence of a 2D convolutional layer with a 7 × 7 × 8 kernel and ReLU activation, a fully connected layer with 2 neurons and ReLU activation, and a Softmax layer serving as the final output layer. The quantified model exhibits a minimal loss of accuracy, with an area under the curve of 0.97 (Figure 13B), and an overall prediction accuracy of 0.92 (Figure 13C). The responder detects seizure onset using the logic flow in Figure 13D and immediately administers midazolam (1 mg/kg). In the experimental group, this first dose reduced seizure frequency within minutes. If the device's AI detects seizure recurrence, it triggers a second midazolam dose, suppressing seizures for the remaining 90 min (Figure 13E). This system builds a complete closed loop of in vivo autonomous sensing‐decoding‐execution: microfluidic devices are responsible for the precise delivery of nanoliter‐level drugs, while local AI is responsible for real‐time decoding of millivolt‐level neural signals at the millisecond level and life‐and‐death decisions. This marks the ultimate leap for microfluidic‐AI systems from continuous monitoring and early warning to fully autonomous intelligent response, providing a revolutionary solution for managing sudden, critical diseases.

**FIGURE 13:**
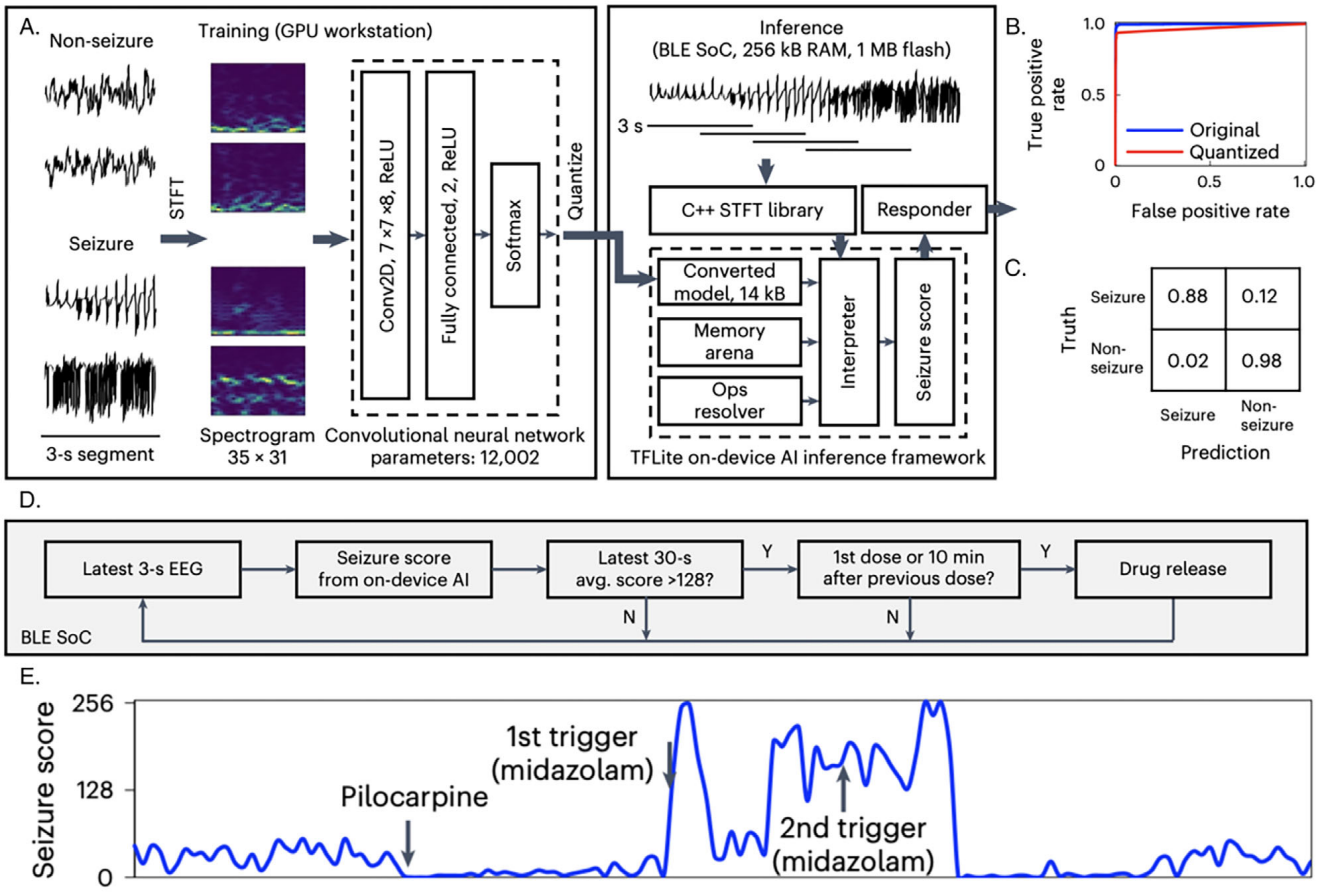
AI‐based, wireless, battery‐less, implantable, real‐time control‐agent release microfluidic device. (A) Training of a seizure detection neural network model and implementation of the quantized model onto the BLE SoC for on‐device AI. (B) Receiver operating characteristic curves of the original and quantized models. (C) Confusion matrix of the on‐device AI model. (D) On‐device algorithm for real‐time closed‐loop management of seizures. (E) In a second rat model, seizures were mitigated by autonomous release of 2 doses of midazolam upon detection of seizures. Reproduced with permission [112]. Copyright 2023, Springer Nature.

In conclusion, AI‐driven analysis and microfluidic‐enabled ultra‐small‐volume decoding are complementary and indispensable pillars. Wearable microfluidics serves as a vital physical interface bridging the human body and the digital healthcare ecosystem. By enabling precise microscale manipulation of biofluids, these systems generate continuous, multimodal physiological data that fuel intelligent algorithms, which in turn guide adaptive therapeutic decisions. This establishes a seamless, closed‐loop cycle of monitoring, analysis, and intervention, integrated into everyday life. AI can analyze patient responses to medication and dynamically adjust treatment plans, enabling personalized medication management. This adaptive capability is especially critical in chronic disease management, where longitudinal monitoring and timely interventions are essential for maintaining clinical stability. The convergence of microfluidics, advanced biosensing, and artificial intelligence represents not merely an incremental advance but a fundamental paradigm shift, paving the way for a future in which healthcare is predictive, preemptive, and deeply personalized.

In summary, transforming this ‘calibration‐decoding‐feedback’ collaborative data pipeline into a clinically validated digital therapy (DTx) or closed‐loop diagnostic and therapeutic platform still requires overcoming multiple translational barriers at the physical, algorithmic, and system levels. As shown in Table 3, different AI architectures inherently involve trade‐offs among model interpretability, dataset scalability, and clinical validation benchmarks. Therefore, systematically addressing these algorithmic shortcomings through AI‐adaptive calibration networks, rigorous uncertainty quantification methods, and multicenter prospective external validation, while strictly adhering to evolving regulatory frameworks for medical device software, represents the ultimate frontier in this field. Successfully navigating this challenge will truly propel intelligent microfluidic biosensing platforms from laboratory prototypes into a precision intervention ecosystem that is approved by national regulatory agencies, highly trusted in clinical practice, and deeply personalized.

**TABLE 3:** AI/ML architectures for biosignal interpretation and their validation benchmarks.

Reference	Architecture	Input signal	Interpretability	Sample/Dataset	Bias mitigation strategy	Performance	Edge AI feasibility
Li et al. [113]	DFT + KNN	RGB Colorimetric array images: Ca^2+^, glucose, uric acid, and K^+^ in sweat	Verified via Density Functional Theory simulation of binding energies	*n* = 180	Supramolecular receptor screening to eliminate cross‐interference.	Ca^2+^ and glucose Acc: 100%, K^+^ Acc: 97.2%, UA Acc: 87.2%	Smartphone‐based semi‐quantitative profiling
Wang et al. [108]	CNN‐GRU	RGB via colorimetric response images;vitamin C, H^+^(pH), Ca^2+^, and proteins in tear	Utilizes predefined database matrix templates for color grading	vitamin C, Ca^2+^, and protein dataset *n* = 2800, pH dataset *n* = 80	Cloud‐assisted database matching to eliminate lighting and ambient noise bias	Pearson's r ≥ 0.983	Cloud server data analysis system
Liu et al. [107]	CNN	RGB via Colorimetric response images; Glucose, pH, and lactate in sweat	Integrates Class Activation Mapping CAM to visualize neural attention	*n* = 4600	Gel capsules isolate indicators; CAM algorithm filters out background noise.	The matching degree with the laboratory measurement values reached 91.0%–99.7%	Optimized CNN deploys on smart terminals.
Dong et al. [114]	Multilayer Perceptron (MLP) auto‐calibration neural network system	Potentiometric voltage; Dimethylamine, Creatinine, Glucose, and H^+^ (pH) in urine	N/A	N/A	MLP adaptive calibration framework map‐corrects potential drift and severe cross‐channel pH interference	Fitting accuracy ≥98%	Seamless edge mobile app system‐level deployment
Xu et al. [115]	Gradient‐boosted decision tree XGBoost, linear regression and SVM	Potentiometric voltage and amperometric current: glucose, lactate, uric acid, sodium ions, potassium ions and ammonium in sweat	N/A	*n* = 60 000	> 100 h continuous dynamic operation against baseline drift	Stress classification Acc: 98.0%, stress regression confidence level: 98.7%	

## 5 Challenges and Future Trends

### 5.1 AI Bias and Validation Frameworks

As microfluidic wearables and implants generate increasingly complex, high‐dimensional biomarker data, the reliance on artificial intelligence for data interpretation intensifies, and with it, the associated risks of algorithmic bias [116]. Bias can arise at multiple stages: from training datasets that inadequately represent diverse populations in terms of age, gender, ethnicity, and comorbidities, to flawed feature selection that may correlate biomarkers with health outcomes in non‐causal or population‐specific ways. In the context of minimal‐volume decoding, where signals are inherently low‐amplitude and noisy, biased algorithms risk misinterpreting individual variations as pathological or, conversely, failing to detect critical signals in underrepresented groups [117]. This threatens the equitable deployment of personalized medicine, potentially exacerbating existing healthcare disparities.

Addressing these algorithmic challenges requires a robust, multi‐layered validation framework, which currently centers on a critical debate: the tension between clinical adoptability and algorithmic performance. Traditionally, clinical practitioners and regulatory bodies argue that transparent and interpretable AI models are crucial for clinical adoption, declaring that completely uninterpretable ‘black‐box’ DL models are untenable in medical diagnostics due to the lack of physiological transparency [118]. However, the rigid demand for intrinsic interpretability often clashes with the engineering realities of biological sensing. The original microfluidic output inherently involves complex physiological interactions, nonlinear sensor drift, and environmental matrix interference, which cannot be captured by a simple and mathematically transparent linear model. To resolve this, medical AI must shift from dogmatically rejecting high‐performing DL toward building empirical trust. When trained on large, standardized clinical datasets, deep neural networks autonomously perform internal self‐calibration. Rather than relying on human‐designed heuristics, these adequately scaled models implicitly correct for baseline drift and demographic variation, transforming the ‘unreliable black box’ into a robust, self‐rectifying predictive engine. To earn clinical trust, the field should adopt post‐hoc explainability tools alongside rigorous uncertainty quantification, which mathematically defines safety boundaries and alerts clinicians to out‐of‐distribution signals.

The execution of these self‐rectifying models further introduces a strategic, architectural debate regarding computational deployment: the trade‐off between centralized cloud computing and localized edge AI deployment. Cloud computing provides virtually unlimited bandwidth to run massive, complex models for high‐dimensional diagnostic profiling, but is inherently limited by data privacy concerns, wireless latency, and a dependence on continuous connectivity. Conversely, local edge deployment onto resource‐constrained micro‐SoCs guarantees the milliseconds‐level, low‐latency processing required for life‐critical, closed‐loop interventions, such as real‐time epileptic seizure suppression, even though model quantization and compression might limit absolute analytical precision.

Ultimately, this computational trade‐off worsens over time due to physiological drift and in vivo biosensor degradation. Since static AI models inevitably degrade under such temporal shifts, the field must shift from pre‐trained networks to continual learning and dynamic domain adaptation. Running these adaptive algorithms on the device enables real‐time weight updates, continuously compensating for sensor ageing and baseline drift without catastrophic forgetting.

Embedding these spatiotemporal AI innovations, especially edge‐cloud hybrid architectures and localised self‐learning, within rigorous prospective clinical trials enables evaluation against gold‐standard diagnostics in diverse, real‐world settings. However, navigating the translational pipeline from clinical trials to market entry requires these adaptive platforms to align with evolving regulatory frameworks for Software as a Medical Device (SaMD) and AI‐enabled Medical Devices (AIaMD) [119, 120]. Traditional static regulatory approvals are fundamentally incompatible with on‐device continual learning, as algorithms are historically required to remain locked post‐approval. To bridge this regulatory gap, emerging pathways such as the FDA's Predetermined Change Control Plan (PCCP) offer a viable solution, allowing manufacturers to pre‐specify protocolized algorithmic updates for sensor drift compensation and physiological adaptation without requiring re‐clearance for every dynamic iteration [121]. Within this framework, establishing clinician trust and securing regulatory approval will hinge not on oversimplified, artificially interpretable models, but on the integration of robust, hardware‐software dual safety guardrails such as physical dosing limits combined with algorithmic uncertainty thresholds, alongside empirically proven reliability and systematic bias mitigation across heterogeneous patient populations.

### 5.2 Biological Interfaces and Material Degradation: The Patient‐Device Hardware Barrier

The translation of microfluidics‐enabled wearables and implantables from laboratory prototypes to sustained clinical adoption is fundamentally bottlenecked by the dynamic, hostile nature of the in vivo and on‐skin biological interfaces. At the skin‐substrate junction, long‐term patient wearability and biosignal fidelity are inextricably linked [122]. Continuous dermal contact with elastomeric substrates such as PDMS and PI often disrupts natural epidermal transpiration, predisposing users to sweat accumulation, localized dermatitis, and contact hypersensitivity. These skin‐substrate micromotion artifacts and localized inflammatory responses do not merely compromise physical comfort and lower user adherence; they fundamentally alter the physical boundary conditions of fluidic sampling and chemical mass transport, generating transient motion artifacts and severe signal drift.

Complicating this interfacial instability is the persistent challenge of biofouling and non‐specific protein adsorption within the microfluidic channels. Over time, active biofouling degrades sensor sensitivity and alters the microchannel hydrodynamic flow rates, necessitating complex antifouling surface modifications or integrated active‐cleaning mechanisms that increase system complexity. When these physicochemical barriers are coupled with the thermodynamic instability of biorecognition elements such as the gradual denaturation of enzymes or antibodies under fluctuating physiological pH, temperature, and mechanical shear, the system experiences unpredictable baseline drift [123, 124]. If users are forced to perform frequent, complex manual calibration protocols to correct this drift, it creates a steep cognitive load and digital literacy barrier, disproportionately marginalizing elderly or tech‐insensitive patient cohorts. Therefore, AI‐adaptive calibration can to some extent help eliminate differences, but the stability of biomaterials and interfaces remains the cornerstone for long‐term wear, and resolving biological interface instability is not merely an engineering issue of material science, but a prerequisite for ensuring long‐term user compliance and preventing the generation of garbage or incomplete datastreams.

### 5.3 System‐Level Energy, Digital Infrastructure, and Regulatory Hurdles: The Scalability Barrier

The translation from laboratory prototype to reliable commercial and clinical product involves overcoming persistent technical barriers. Biofouling and non‐specific adsorption in microchannels degrade sensor performance over time, necessitating the use of advanced antifouling materials or self‐cleaning mechanisms. Power constraints remain a fundamental limit; while energy harvesting, such as piezoelectric [125], triboelectric, and biofuel cells, shows promise, achieving stable, sufficient power for continuous sensing, computation, and communication across all usage scenarios remains unproven at scale. Manufacturing scalability presents another critical challenge. Reproducibly fabricating devices with micron‐scale features, ensuring sensor‐to‐sensor calibration consistency, and doing so cost‐effectively are prerequisites for mass production.

Even with a perfectly bio‐compatible and stable physical interface, scaling microfluidic technologies to broader clinical workflows faces immense system‐level energy, computational, and regulatory bottlenecks. First, power constraints remain a fundamental physical limit. While microscale energy harvesting such as piezoelectric, triboelectric, and enzymatic biofuel cells offers an elegant theoretical solution, generating stable, continuous power sufficient for simultaneous microfluidic pumping, Edge AI with on‐device computing and continuous wireless transmission across dynamic everyday usage scenarios remains unproven at scale. Consequently, devices are forced to balance sampling frequency against battery longevity, introducing critical temporal data gaps.

This energy‐bandwidth trade‐off directly impacts data integration with existing healthcare legacy systems [126]. The continuous, high‐frequency, and heterogeneous multi‐analyte datastreams generated by microfluidic wearables are fundamentally incompatible with traditional Electronic Health Record (EHR) frameworks, which are designed historically for episodic, highly structured clinical inputs. Direct, raw data streaming risks overloading clinical databases and precipitating severe alarm fatigue among practitioners, who require actionable, synthesized diagnostic alerts rather than continuous fluctuating telemetry. Overcoming this systemic bottleneck requires the deployment of standardized, secure, and interoperable data pipelines—such as the Fast Healthcare Interoperability Resources (FHIR) protocol linked with low‐power on‐device AI that compresses raw data into high‐fidelity diagnostic trends prior to transmission. Finally, translating these highly integrated, software‐hardware‐coupled combination systems through the evolving regulatory pathways, such as the FDA and EMA, remains a protracted hurdle. Regulatory bodies demand rigorous validation of not only sensor‐to‐sensor manufacturing calibration consistency at a mass‐production scale but also the clinical safety, cybersecurity, and ethical interpretability of the embedded decision‐making algorithms, representing the final frontier for digital health protection.

### 5.4 Outlook and Convergent Trends

Despite existing technical and translational challenges, the field's trajectory points toward microfluidic wearables and implants that are increasingly intelligent, autonomous, and seamlessly integrated into daily life. Several convergent trends are shaping this next generation of systems. First, the incorporation of embedded AI and edge‐computing architectures is expected to shift data processing from cloud‐dependent pipelines to on‐device analytical engines, enabling real‐time, low‐latency decision‐making that supports closed‐loop therapeutic responses while enhancing data privacy [107, 108, 113]. Parallel advances in energy autonomy, including motion‐based harvesting, thermal gradients, and biochemical energy conversion, aim to provide long‐term or even indefinite device operation without reliance on bulky external power sources [127].

Convergence with nanomaterials, soft robotics, and next‐generation telecommunications will further extend functional capabilities, allowing highly sensitive analyte detection, targeted micro‐actuation at the cellular scale, and robust remote patient monitoring through high‐bandwidth, low‐latency connectivity [128]. In parallel, progress in scalable manufacturing technologies, such as roll‐to‐roll printing, high‐throughput micromolding, and advanced 3D printing, is paving the way toward cost‐effective mass production. These manufacturing advances are essential not only for commercial deployment but also for global health implementation, where the portability and lab‐on‐a‐chip functionality of microfluidic systems could substantially expand access to advanced diagnostics in resource‐limited settings [129].

Looking ahead, continuous, longitudinal physiological datasets generated by these platforms will serve as the foundation for predictive and preventive health models, including AI‐driven digital twins capable of identifying pre‐symptomatic risks, forecasting disease trajectories, and personalising intervention strategies at scale [130, 131]. Such developments signal a fundamental shift from reactive healthcare to proactive, precision public health.

Realizing this vision of microfluidics‐enabled personalized medicine, however, requires coordinated advances across technical, clinical, ethical, and regulatory domains. Addressing challenges related to algorithmic bias, reproducibility, clinical validation, user‐centric design, and scalable manufacturing will be critical for ensuring that these technologies are not only effective but also equitable, trustworthy, and widely adoptable. By confronting these multidimensional barriers, the field can unlock its full transformative potential and advance toward a healthcare paradigm that is predictive, personalised, preventive, and deeply integrated into everyday life.

## 6 Conclusion

This review underscores microfluidics as a pivotal enabling technology for the next generation of wearable and implantable health systems, facilitating continuous, multiplexed, and real‐time biomarker analysis from minute volumes of biofluid. The convergence of flexible materials, advanced biosensing, and AI‐driven analytics is reshaping personalised medicine, transitioning healthcare from episodic to continuous, adaptive management. However, the path to clinical translation is fraught with multifaceted challenges. Beyond enduring technical barriers such as power autonomy, manufacturing scalability, and long‐term biocompatibility, we highlight a critical yet often overlooked translational gap: the vulnerability of AI models to bias stemming from data heterogeneity across biofluid sources, patient populations, and sensing platforms. Without standardized datasets and robust validation frameworks, algorithmic decisions risk perpetuating healthcare disparities and compromising diagnostic reliability.

Future progress must therefore adopt a dual focus: advancing device performance while embedding computational fairness into the development pipeline. This entails establishing reproducible data pipelines, implementing rigorous clinical validation protocols, and fostering interoperability with electronic health records. Additionally, ethical considerations, including data privacy, liability in autonomous systems, and user‐centric design for comfort and accessibility, must be integral to system deployment. As these challenges are addressed through collaborative efforts among engineers, clinicians, and regulators, microfluidics‐enabled systems will evolve from promising prototypes to indispensable tools for predictive, preventive, and precisely personalized healthcare, ultimately enhancing health outcomes across diverse global populations.

## Conflicts of Interest

The authors declare no conflicts of interest.

## Data Availability

Data sharing not applicable to this article as no datasets were generated or analysed during the current study.
